# *APOE* genotype influences the gut microbiome structure and function in humans and mice: relevance for Alzheimer’s disease pathophysiology

**DOI:** 10.1096/fj.201900071R

**Published:** 2019-04-08

**Authors:** Tam T. T. Tran, Simone Corsini, Lee Kellingray, Claire Hegarty, Gwénaëlle Le Gall, Arjan Narbad, Michael Müller, Noemi Tejera, Paul W. O’Toole, Anne-Marie Minihane, David Vauzour

**Affiliations:** * Alimentary Pharmabiotic Centre (APC) Microbiome Ireland, University College Cork, Cork, Ireland;; † School of Microbiology, University College Cork, Cork, Ireland;; ‡ Norwich Medical School, University of East Anglia, Norwich, United Kingdom; and; § Quadram Institute Bioscience, Norwich Research Park, Norwich, United Kingdom

**Keywords:** apolipoprotein E, gut microbiota, metabolomics, butyrate, SCFAs

## Abstract

Apolipoprotein E (*APOE*) genotype is the strongest prevalent genetic risk factor for Alzheimer’s disease (AD). Numerous studies have provided insights into the pathologic mechanisms. However, a comprehensive understanding of the impact of *APOE* genotype on microflora speciation and metabolism is completely lacking. In this study, we investigated the association between *APOE* genotype and the gut microbiome composition in human and *APOE*–targeted replacement (TR) transgenic mice. Fecal microbiota amplicon sequencing from matched individuals with different *APOE* genotypes revealed no significant differences in overall microbiota diversity in group-aggregated human *APOE* genotypes. However, several bacterial taxa showed significantly different relative abundance between *APOE* genotypes. Notably, we detected an association of *Prevotellaceae* and *Ruminococcaceae* and several butyrate-producing genera abundances with *APOE* genotypes. These findings were confirmed by comparing the gut microbiota of *APOE*-TR mice. Furthermore, metabolomic analysis of murine fecal water detected significant differences in microbe-associated amino acids and short-chain fatty acids between *APOE* genotypes. Together, these findings indicate that *APOE* genotype is associated with specific gut microbiome profiles in both humans and *APOE*-TR mice. This suggests that the gut microbiome is worth further investigation as a potential target to mitigate the deleterious impact of the *APOE4* allele on cognitive decline and the prevention of AD.—Tran, T. T. T., Corsini, S., Kellingray, L., Hegarty, C., Le Gall, G., Narbad, A., Müller, M., Tejera, N., O’Toole, P. W., Minihane, A.-M., Vauzour, D. *APOE* genotype influences the gut microbiome structure and function in humans and mice: relevance for Alzheimer’s disease pathophysiology.

The gut microbiome is intimately involved in numerous aspects of human physiology. Emerging evidence links perturbations in the microbiome to neurodegeneration and Alzheimer’s disease (AD), with (neuro)inflammation proposed as an etiological link (1–3).

The extent to which host genetic variation determines the microbiome composition is still currently debated. Indeed, although previous studies have reported that the microbiomes of humans and mice are associated with host genetic variation (4) and have identified several heritable bacterial taxa (5–7), other studies have reported a stronger environmental influence compared with host genetics in shaping human gut microbiota (8). Thus, the extent to which human genetics shape microbiome composition remains unclear.

Apolipoprotein E (*APOE*) genotype is the strongest prevalent risk factor for neuropathology and AD (9–11). ApoE was originally identified as a component of systemic circulating lipoproteins and a member of a family of apolipoprotein modulators of their metabolism. It has subsequently emerged as the almost exclusive lipid transporter in the CNS (12, 13). In humans, APOE exists in 3 different isoforms (apoE2, apoE3 and apoE4), arising from 3 different alleles (ε2, ε3, and ε4). These alleles give rise to 3 homozygous (*APOE2/E2*, *APOE3/E3* and *APOE4/E4*) and 3 heterozygous (*APOE3/E2*, *APOE4/E3* and *APOE4/E2*) genotypes in humans (14). Generally, 50–70% of populations present with the *APOE3/E3* genotype, with the ε3 allele accounting for 70–80% of the gene pool, and the ε2 and ε4 alleles accounting for 5–10% and 10–15%, respectively (14). *APOE4* carrier status is highly predictive of dementia and AD, with *APOE3/E4* and *APOE4/E4* being at 3–4- and 8–12-fold increased risk and a much earlier age of onset (9). Although the etiological basis of *APOE4*-neuropathological associations has been widely researched and reported, the main etiological mechanism has not been clearly defined. The ApoE protein is involved in multiple biologic processes, including lipoprotein metabolism (15), intracellular cholesterol utilization (16), cell growth (17), immunoregulation, (neuro)inflammation (18, 19), and neuroprotection (20). Although the role of ApoE in gut chylomicron metabolism and in inflammation has been described, and gut microbiota modulation improves the cardio-metabolic profile in ApoE-deficient mice (*Apoe^−/−^*) (21), the impact of *APOE* genotype on intestinal integrity and gut microbiome composition and metabolism is currently unknown (22).

In the present study, we explore the hypothesis that *APOE* variation influences the microbiome composition and its subsequent metabolism. Our experiments using human fecal samples and *APOE*–targeted replacement (TR) mice revealed significantly different relative abundance between bacterial taxa according to *APOE* genotypes. Furthermore, using a metabolomic approach, differences in microbe-associated amino acids and short-chain fatty acids (SCFAs) according to *APOE* genotypes were also observed. Taken together, our findings indicate that *APOE* genotype associates with specific gut microbiome profiles, which may affect the host metabolism and ultimately contribute to AD pathology.

## MATERIALS AND METHODS

### Ethics approval and consent to participate

The research involving human material has been performed in accordance with the Declaration of Helsinki. The study protocols were approved by the National Research Ethics Service Committee [13/EE/0066 for the chocolate, orange juice, and blackberry (COB) study, NCT01922869; 14/EE/0189 for the Cognitive Ageing, Nutrition, and Neurogenesis (CANN) study, NCT02525198], and all participants consented to provide stool samples and to the use of the stored samples for research purposes.

All experimental procedures and protocols involving animals were reviewed and approved by the Animal Welfare and Ethical Review Body and were conducted within the provisions of the Animals (Scientific Procedures) Act of 1986 (Reference 70/8710).

### Participants, sample collection, *APOE* genotyping, and biochemical analysis

Fifty-six healthy participants, aged between 56 and 78 yr, were prospectively selected according to *APOE* genotype from the COB (NCT01922869) and the CANN (NCT02525198) studies for the analysis of their gut microbiota speciation. Participants were provided with fecal collection kits, which included a stool collection bag and an ice pack. They were asked to defecate directly into the bag, which was secured and placed with the ice pack into an insulated container and delivered to the study scientist. The samples were then homogenized by physical manipulation before aliquots were taken and stored at −80°C.

*APOE* genotyping was carried out as previously described (23). Briefly, DNA was isolated from the buffy coat layer of 8 ml of blood collected into sodium heparin mononuclear cell preparation tubes with the use of the Qiagen DNA blood mini kit (Qiagen, Germantown, MD, USA). Allelic discrimination of the *APOE* gene variants was conducted with TaqMan PCR technology (7500 Instrument; Thermo Fisher Scientific, Waltham, MA, USA) and Assay-on-Demand single nucleotide polymorphism genotyping assays (Thermo Fisher Scientific). The *APOE* haplotypes (E2/E3, E3/E3, E3/E4, and E4/E4) were determined from the alleles for the *APOE* single nucleotide polymorphisms rs7412 and rs429358. Twenty-four participants were selected as *APOE4* carriers (*APOE*3/4 and *APOE*4/4; 12 men and 12 women), with 32 participants selected as *APOE4* noncarriers (*APOE*2/3 and *APOE*3/3; 16 men and 16 women), with the selection process matching the genotype groups for age, body mass index (BMI), and gender.

Serum LPS binding protein (LBP) (ab213805; Abcam, Cambridge, MA, USA) and haptoglobin (ab108856; Abcam) plasmatic concentrations were detected by ELISA kits according to the manufacturer’s instructions. The assay range for the LBP and the haptoglobin ELISA kits was 1.56–100 and 0.078–20 µg/ml, respectively. Serum samples were diluted until the LBP or haptoglobin concentrations were in the range of these kits.

### Human fecal bacterial DNA extraction and 16S rRNA amplicon sequencing

Total genomic DNA were isolated from human fecal samples using DNeasy Blood and Tissue Kit (Qiagen) following the manufacturer’s instructions with some modifications following the repeated bead-beating method (24). The V3–V4 hypervariable region of the 16S rRNA gene was amplified to generate a fragment of 460 bp using the forward primer: 5′-TCGTCGGCAGCGTCAGATGTGTATAAGAGACAGCCTACGGGNGGCWGCAG-3′, and reverse primer: 5′-GTCTCGTGGGCTCGGAGATGTGTATAAGAGACAGGACTACHVGGGTATCTAATCC-3′ (25). The Illumina overhang adapter sequences (Illumina, San Diego, CA, USA) were added to the 16S rRNA gene specific primer sequences. Each 30 µl PCR reaction contained 10 ng/µl microbial genomic DNA, 0.2 µM of each primer, 15 µl of 2× Phusion Taq High-Fidelity Mix, and 10.6 µl of nuclease-free water. The PCR conditions were initial denaturation 98°C for 30 s; 25 cycles of 10 s at 98°C, 15 s at 55°C, and 20 s at 72°C; and 72°C for 5 min for final elongation. The SpriSelect Reagent Kit (Beckman Coulter, Brea, CA, USA) was used to purify the amplicons. The Qubit double-stranded DNA High Sensitivity Assay Kit (Thermo Fisher Scientific) was followed for quantification and pooling. Library preparation was carried out by Teagasc (Oak Park, Ireland) on the Illumina MiSeq platform using paired-end Illumina sequencing run (2 × 250 bp).

### *APOE*-TR mice

Twenty young (4 mo; *n* = 10/genotype) and 12 old (18 mo; *n* = 6 per genotype) male human *APOE3* [B6.129P2-Apoe^tm2(APOE*3)Mae^ N8] and *APOE4* [B6.129P2-Apoe^tm2(APOE*4)Mae^ N8] TR mice homozygous for the human *APOE3* or *APOE4* gene (Taconic Farms, Germantown, NY, USA) were used in these experiments (*n* = 10/genotype) (26). The model was created by Dr. Maeda (University of North Carolina, USA) by targeting the murine *APOE* gene for replacement with the human *APOE3* and *APOE4* allele in E14TG2a embryonic stem cells and injecting the targeted cells into blastocysts. Resultant chimeras were backcrossed to C57BL/6 for 8 generations (N8). Mice were housed 2 per cage and were maintained in controlled environment (21°C; 12-h light/dark cycle; light from 7:00 am) and fed a standard chow diet (RM3-P; Special Diets Services, Witham, United Kingdom) for the duration of the experiments.

### Mice genomic DNA extraction and 16S rRNA amplicon sequencing

Bacterial genomic DNA was extracted from fecal samples using a FastDNA SPIN Kit for Soil (MP Biomedicals, Santa Ana, CA, USA) with 3 bead-beating periods of 1 min (27). Bacterial DNA concentration was normalized to 1 ng/μl by dilution with DNA elution solution (MP Biomedicals) to produce a final volume of 20 μl. Normalized DNA samples were sent to the Centre for Genomic Research (Liverpool, United Kingdom) for PCR amplification of the 16S rRNA gene and paired-end Illumina sequencing (2 × 250 bp) on the MiSeq platform. The V4 region of the 16S rRNA gene was amplified to generate a 254-bp insert product as previously described (28). The first round of PCR was performed using the forward primer: 5′-ACACTCTTTCCCTACACGACGCTCTTCCGATCTNNNNNGTGCCAGCMGCCGCGGTAA-3′, and the reverse primer: 5′-GTGACTGGAGTTCAGACGTGTGCTCTTCCGATCTGGACTACHVGGGTWTCTAAT-3′, which include recognition sequences that enable a second nested PCR, using the N501f and N701r primers, to incorporate Illumina adapter sequences and barcode sequences. The use of these primers enables efficient community clustering for the length of reads obtained through Illumina sequencing, and this method also allows for high-throughput sequencing. Sequencing data were supplied in fastq format with adaptors already trimmed.

### Metabolomic analyses

Metabolites were analyzed and quantified by [^1^H] NMR analysis. The preparation method was similar to that previously described (29–32). Fecal contents were extracted from mice caeca and prepared for [^1^H] NMR using procedures validated and published in our laboratory for fecal metabolomics (33–35). Briefly, 20 mg of frozen fecal materials were thoroughly mixed on a vortex with 1 ml of saline phosphate buffer [1.9 mM Na_2_HPO_4_, 8.1 mM NaH_2_PO_4_, 150 mM NaCl (MilliporeSigma, Burlington, MA, USA)], and 1 mM trimethylsilylpropanoic acid [sodium 3-(trimethysilyl)-propionate-d4] in deuterated water (Goss Scientifics, Crewe, United Kingdom), followed by centrifugation (18,000 *g*, 1 min). Supernatants were removed, filtered through 0.2 μm Fluoropore polytetrafluoroethylene membrane filters (MilliporeSigma), and stored at −20°C until required.

After mixing and centrifugation, 500 µl was transferred into a 5-mm NMR tube for spectral acquisition. High resolution [^1^H] NMR spectra were recorded on a 600-MHz Bruker Avance spectrometer fitted with a 5-mm TCI proton-optimized triple resonance NMR inverse cryoprobe and a 60-slot autosampler (Bruker, Billerica, MA, USA). Sample temperature was controlled at 300 K. Each spectrum consisted of 128 scans of 32,768 complex data points with a spectral width of 14 ppm (acquisition time 1.95 s). The noesypr1d presaturation sequence was used to suppress the residual water signal with low power selective irradiation at the water frequency during the recycle delay (D1 = 2 s) and mixing time (D8 = 0.15 s). A 90° pulse length of 8.8 μs was set for all samples. Spectra were transformed with a 0.3-Hz line broadening and zero filling, manually phased, baseline corrected, and referenced by setting the trimethylsilylpropanoic acid methyl signal to 0 ppm. Metabolites were identified using information found in the literature or on the Human Metabolome Database (*http://www.hmdb.ca/*), and by use of the 2-dimensional NMR methods, [^1^H]-[^1^H] correlation spectroscopy, [^1^H]-[^13^C] heteronuclear single quantum correlation, and [^1^H]-[^13^C] heteronuclear multiple bond correlation spectroscopy (36) and quantified using the software Chenomx NMR Suite 7.0 (Supplemental Fig. S1). Details on parameter settings for 2-dimensional NMR can be found in Le Gall (37).

### Analysis of 16S amplicon sequencing data from humans and mice

Bioinformatics analysis of 16S amplicon sequencing data from humans and mice were performed using the Quantitative Insights into Microbial Ecology (QIIME) v.1.9.1 (38) and uSearch v.8.1 (39) software and the following procedure. First, the paired-end reads were merged using Fast Length Adjustment of Short Reads (FLASH) v.1.2.8 (40); then, adaptors were removed from reads using cutadapt v.1.8.3 (41). The sequences were demultiplexed and filtered using QIIME with the split_libraries_fastq.py script; all reads with a quality score below 19 were removed. Reverse primers were removed using QIIME with the truncate_reverse_primer.py script. An operational taxonomic unit (OTU) table was obtained using uSearch. Unique sequences were filtered (derep_fulllength) and sorted by length (sortbylength) with a length of 373–473 nt for the V3–V4 region and a length of 237–271 nt for the V4 region. After singleton removal (sortbysize), the remaining sequences were clustered into OTUs at a default 97% sequence identity (cluster_otus) and filtered for chimeras against the ChimeraSlayer reference database (uchime_ref) (42). All sequences were mapped against this database (usearch_global) to generate an OTU table. Classification of representative sequences for each OTU was carried out using mothur v.1.36.1 (43) against the 16S rRNA reference of Ribosomal Database Project database trainset 14 (44). To ensure an even sampling depth, we used QIIME to generate rarefied OTU tables with the single_rarefaction.py script and to compute α diversity metrics (chao1, phylogenetic diversity, Shannon’s diversity index, evenness) with the alpha_rarefaction.py script and β diversity metrics (weighted UniFrac, unweighted UniFrac, and Bray-Curtis distances) with the beta_diversity.py script.

### Statistical analysis

Statistical analysis was carried out using R v.3.5.1 software packages (45). The significant differences in clinical measures, α diversity, and abundances of each taxonomic unit between ≥2 groups were detected using the Mann-Whitney *U* test or Kruskal-Wallis *H* test with Dunn’s multiple comparison test, respectively. The *P* values were corrected for multiple testing by Benjamini-Hochberg correction to control false discovery rate. Differences in β diversity were determined using permutational multivariate ANOVA (PERMANOVA) (vegan R package; *https://cran.r-project.org/package=vegan*).

Multivariate statistical analysis (sparse partial least squares discriminant analysis and metabolite set enrichment analysis) of the [^1^H] NMR data was carried out using the MetaboAnalyst R package v.1.0.0 (*https://www.metaboanalyst.ca/*). Coinertia analysis (COIA) was used to investigate the relationships between the fecal metabolome and the composition of microbiota at OTU level using the coinertia function in the ade4 R package (46). Only OTUs present in at least 50% of the samples were used in COIA. Overall similarity in the structure between 2 data sets were measured by *RV* coefficient. The significance of the *RV* coefficient was tested using the Monte Carlo permutation test (47).

## RESULTS

### Descriptive statistics of human *APOE*-genotyped cohorts

A total of 56 fecal samples were analyzed from participants of the COB (NCT01922869) and the CANN studies (NCT02525198) (Norwich Clinical Centre, Norwich, United Kingdom) with the 4 *APOE* genotype groups selected to be matched for sex, age, and BMI (**Table 1** and Supplemental Table S1).

**TABLE 1:** Clinical characteristics of participants according to APOE genotypes

Parameter	*E2/E3*	*E3/E3*	*E3/E4*	*E4/E4*	*P**^a^*
*n*	14	18	18	6	
Age (yr)	68.6 ± 4.6	68.5 ± 5.0	68.6 ± 3.0	67.7 ± 6.1	1.0
Sex [male:female (*n*)]	7:7	9:9	9:9	3:3	1.0
BMI (kg/m^2^)	25 ± 2.2	26.3 ± 2.6	26.1 ± 3.1	25 ± 1.9	0.42

Data presented as means ± sd.

a Significance was calculated by the Kruskal-Wallis *H* test.

### Difference in human gut microbiota composition between *APOE* genotypes

The V3–V4 hypervariable region of the 16S rRNA gene was PCR amplified from fecal samples collected from participants to generate an amplicon of ∼460 bp. Sequencing this amplicon allows determination of microbiota composition. The reads were clustered at a 97% similarity threshold into 3314 unique OTUs or sequence-based bacterial classification, approximating to species. The total OTUs were assigned to 15 phyla, 27 classes, 43 orders, 70 families, and 155 unique genera across the entire data set. The vast majority (99.5%) of all sequences were affiliated to 5 dominant phyla, mainly in the *Firmicutes* (82.2 ± 10.8%), with lower assignment to phyla *Bacteroidetes* (7.7 ± 6.3%), *Actinobacteria* (3.8 ± 4.5%), *Proteobacteria* (3.2 ± 9.5%), and *Verrucomicrobia* (2.6 ± 5.1%) (Supplemental Fig. S2*A*). After rarefaction with a depth of 8736 reads per sample, α diversity (net diversity within a single sample/subject) was measured by calculating 3 diversity indices, namely, chao1 (richness), phylogenetic diversity, and Shannon diversity index. None of these metrics was significantly different between *APOE* genotypes (Supplemental Fig. S3*A*). Similarly, there was no significant difference in any of the α diversity metrics between males and females (Supplemental Fig. S3*B*). However, the microbiota α diversity of obese subjects (*n* = 3) was significantly lower than that in normal weight and overweight subjects (*P* < 0.05, Supplemental Fig. S3*C*), in line with previous observations (48). β diversity analysis (which measures interindividual microbiota relatedness) was performed using principal coordinates analysis (PCoA) clustering based on unweighted and weighted UniFrac distances. A PERMANOVA test was employed for testing associations between clinical parameters and microbiota composition and results are given in Supplemental Table S2. There was no difference in β diversity of gut microbiota composition according to *APOE* genotype (Supplemental Fig. S4*A*). However, we observed a weak but significant association between microbiota composition and gender and BMI categories (Supplemental Fig. S4*B*, *C*).

**Figure 1:**
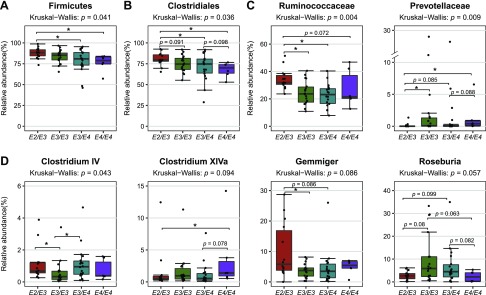
Box plot of the relative abundance distribution of selected taxa at phylum level (*A*), order level (*B*), family level (*C*), and *Clostridium* cluster or genus level (*D*). Significant difference was observed in selected bacterial taxon abundance between human *APOE* genotypes. Significance values were calculated by the Kruskal-Wallis *H* test for all genotypes, followed by Dunn’s multiple comparisons and adjusted for false discovery rate using the Benjamini-Hochberg correction. *P < 0.05.

Although α and β diversity analyses of the gut microbiota did not discriminate between *APOE* genotypes, these are global measures that detect relatively large differences in microbiota structure. We therefore questioned whether the relative abundance of any taxa might differ between these genotypes, using the Kruskal-Wallis *H* test to compare all taxa at various phylogenetic assignment levels across all genotypes. We observed that the relative abundance of the phylum *Firmicutes* and order *Clostridiales* was higher in subjects of the *APOE2/E3* genotype than in *APOE3/E4* or *APOE4/E4* (*P* < 0.05; **Fig. 1** and Supplemental Table S3). Furthermore, at the bacterial family level, the abundance of *Ruminococcaceae* (a family of fermentative anaerobes associated with fiber degradation and SCFA production) was higher in *APOE2/E3* than in *APOE3/E3* (*P* = 0.004), *APOE3/E4* (*P* = 0.002), or *APOE4/E4* (*P* = 0.072). On the other hand, the abundance of *Prevotellaceae* was lower in *APOE2/E3* than the other 3 *APOE* genotypes (*APOE3/E3*, *P* = 0.008; *APOE3/E4*, *P* = 0.085; *APOE4/E4*, *P* = 0.015) and was slightly more abundant at close to significant levels (*P* = 0.088) in *APOE3/E4* compared with *APOE4/E4* with mean of relative abundance of 1.79 *vs.* 1.40% (Fig. 1*C* and Supplemental Fig. S2*B* and Supplemental Table S3). Within the *Ruminococcaceae* family, 3 genera, including *Clostridium* cluster IV, *Clostridium* cluster XIVa, and *Gemmiger*, were statistically significant and differentially abundant according to *APOE* genotypes. The abundance of *Clostridium* cluster IV was lower in *APOE3/E3* than in *APOE2/E3* (*P* = 0.027) and *APOE4/E4* (*P* = 0.039), whereas the abundance of *Clostridium* cluster XIVa was higher in *APOE4/E4* than in *APOE2/E3* (*P* = 0.044) and *APOE3/E4* (*P* = 0.078). Higher presence of *Gemmiger* was observed in fecal samples from *APOE2/E3* compared with *APOE3/E3* (*P* = 0.0499) and *APOE3/E4* (*P* = 0.086). Moreover, we observed a higher abundance of *Roseburia* in fecal samples at close to significant levels (*P* < 0.1) in *APOE3/E3* compared with 3 other *APOE* genotypes and in *APOE3/E4* compared with *APOE4/E4* (Fig. 1*D* and Supplemental Fig. S2*C* and Supplemental Table S3).

To determine possible associations between *APOE* genotypes and microbial translocation, we measured the plasma levels of 2 biomarkers of intestinal integrity, namely, haptoglobin and LBP. No significant differences were observed in the levels of haptoglobin and LBP (Supplemental Table S1) according to genotype. Furthermore, no significant correlation was observed between the clinical parameters and both the weighted and unweighted UniFrac distances (Supplemental Table S2).

### Difference in murine gut microbiota composition between *APOE* genotypes

We next sought to investigate if *APOE* genotype–gut microbiota interactions in humans were evident in human transgenic homozygous *APOE3-* and *APOE4-*TR mice at 4 mo (young) and 18 mo (old) of age. Considering the average lifespan of laboratory mice, 18 mo of age would correspond to about 65 human years (49), which approximates our human participants’ age (68.5 ± 4.4 yr).

**Figure 2:**
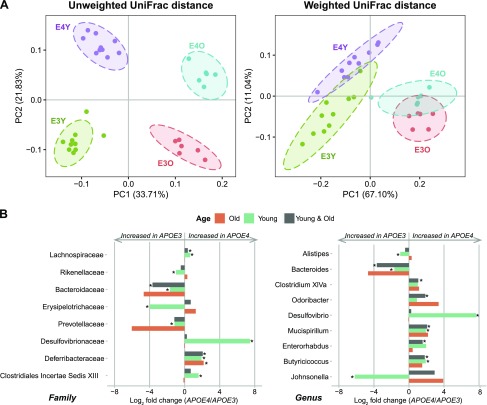
Differences in gut microbiome composition between *APOE3*-TR and *APOE4*-TR mice. *A*) Principal coordinates analysis (PCoA) based on unweighted and weighted UniFrac distances of partial sequences of bacterial 16S rRNA genes showing gut microbiota β diversity grouped by age and *APOE* genotypes. Samples are projected onto the first (PC1) and second (PC2) principal coordinate axes. β diversity analysis reveals significant gut microbiota differences between *APOE*3 and *APOE*4 genotype transgenic mice. Significant differences between groups were calculated by PERMANOVA tests. *B*) Comparison of relative abundance taxa between *APOE3* and *APOE4* in young mice samples, old mice samples, and both age groups combined were represented by log_2_ fold changes. Significant differences in relative abundance of gut microbiota at the family and genus levels between *APOE*3-TR and *APOE*4-TR mice were detected in young mice, old mice, and in combined analysis of young and old mice. Statistical significances were determined by the Mann-Whitney *U* test and were corrected for the multiple comparison using the Benjamini-Hochberg adjustment. E3Y, *APOE3* young mice; E4Y, *APOE4* young mice; E3O, *APOE3* old mice; E4O, *APOE4* old mice. *P < 0.05.

Their gut microbial communities were analyzed based on sequencing the V4 hypervariable region (∼254 bp) of the 16S rRNA gene. There was no significant difference in α diversity between *APOE3* and *APOE4* genotypes. However, in line with our previous studies of microbiota in ageing humans (50) and rodents (51), both chao1 and phylogenetic diversity were much higher in young mice compared with old mice (*P* < 0.001 and Supplemental Fig. S5). Moreover, UniFrac distances (unweighted and weighted) PCoA showed that fecal microbial profiles of young mice separated significantly from those of old mice (PERMANOVA, *P* = 0.001; **Fig. 2*A***). Within each age group, both UniFrac measures showed significant microbiota differences between *APOE3* and *APOE4* genotypes, with the *P* value from PERMANOVA analysis <0.005 (Fig. 2*A*). These differences could be explained by differences detected in the relative abundance of dominant taxa, of which the most dominant were *Firmicutes* (62.8 ± 14.4%) and *Bacteroidetes* (32.3 ± 14.8%), followed by *Proteobacteria*, *Verrucomicrobia*, and *Deferribacteres* accounting for <5% in total (Supplemental Fig. S6).

Analysis of differentially abundant taxa between *APOE3*-TR and *APOE4*-TR animals at the phylum level revealed that *Deferribacteres* in combined young and old mice were notably higher in the *APOE4*-TR mice compared with the *APOE3*-TR mice, whereas the opposite was true for *Candidatus Saccharibacteria*. In addition, lower relative abundance of *Proteobacteria* were seen in *APOE4*-TR young mice when compared with the *APOE3*-TR young mice (Supplemental Fig. S7 and Supplemental Table S4). Although no significant difference was found in aggregated *Firmicute* or *Bacteroidetes* phylum abundance between *APOE* genotypes, we observed an increase in *Firmicutes*:*Bacteroidetes* ratio in old mice when compared with young mice (*P* < 0.001; Supplemental Fig. S8), in agreement with a previous C57BL/6N mouse study (52). At the order level, *Deferribacterales* abundance in combined age groups was significantly higher in the *APOE4*-TR mice compared with the *APOE3*-TR mice. Additionally, *Clostridiales*, *Erysipelotrichales*, and *Desulfovibrionales* in young mice were significantly different in relative abundance between the 2 *APOE* genotypes. The increase of *Lachnospiraceae* and *Deferribacteraceae* abundance and decrease of *Bacteroidaceae* abundance at family level in *APOE4* transgenes compared with *APOE3* was detected in combined age groups. *Desulfovibrionaceae*, *Clostridiales Incertae Sedis* XIII, *Rikenellaceae*, *Prevotellaceae*, and *Erysipelotrichaceae* were also found to be significantly different between *APOE* genotypes in young mice (Fig. 2*B*, Supplemental Fig. S7, and Supplemental Table S4). Those differentially abundant families by *APOE* genotype were reflected by *Mucispirillum*, *Clostridium* cluster XIVa, *Butyricicoccus*, *Odorobacter*, *Enterorhabdus*, and *Bacteroides* in combined age groups and by *Mucispirillum*, *Desulfovibrio*, *Butyricicoccus*, *Bacteroides*, *Alistipes*, and *Johnsonella* in young mice at the genus level (Fig. 2*B*, Supplemental Fig. S7, Supplemental Table S4).

### Fecal metabolite associations with *APOE* genotype and age

**Figure 3:**
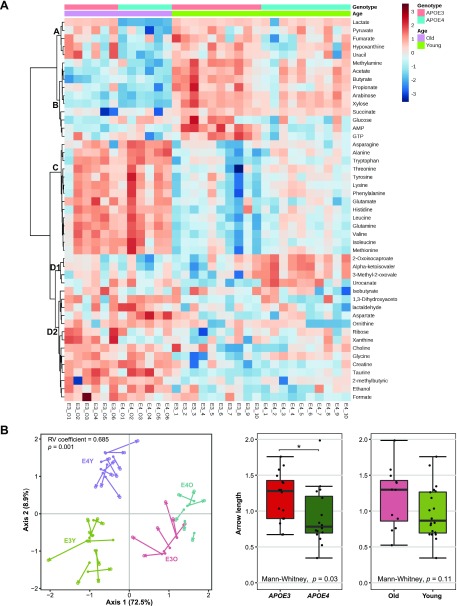
Fecal metabolome analysis of *APOE3*-TR and *APOE4*-TR mice. *A*) Heatmap and cluster analysis of 2-way ANOVA of significantly differentially abundant metabolites grouped by age and *APOE* genotype. Four clusters within significantly differentially abundant metabolites showed distinct *APOE* genotype and age correlations. Clustering was obtained following similarity analysis using the Ward hierarchical algorithm and Euclidean distance metrics. *B*) COIA of the association between metabolites and microbiota composition in the gut. The left panel shows the COIA of the microbiota principal component analysis (solid circle) at OTU level and the principal component analysis of metabolomics (empty circle); length of arrow indicates the divergence between 2 data sets. The right panel shows coinertia of metabolome and microbiota data, represented by arrow length between the 2 data points per sample, grouped according to *APOE* genotype or age. A high overall similarity was found in the structure between the 2 data sets, which was statistically significant, and a higher concordance was found between microbiota composition and metabolites of *APOE4* mice compared with *APOE3* mice. Length of arrow was estimated using Euclidean distance measurement. E3Y, *APOE3* young mice; E4Y, *APOE4* young mice; E3O, *APOE3* old mice; E4O, *APOE4* old mice. *P < 0.05.

In order to improve our understanding of the relationships between metabolite and microbiota composition in the gut, we performed metabolomic analyses of fecal water prepared from caecal contents. Sparse partial least squares discriminant analysis (sPLS-DA) showed a trend for separation according to age and *APOE* genotypes (Supplemental Fig. S9). Two-way ANOVA was therefore performed to investigate interactions between age and *APOE* genotype. Seven metabolites, AMP, α ketoisovaleric acid, glucose, glycine, lactate, oxocaproate, and xanthine were present at significantly different levels in age-*APOE* genotype interaction, whereas 39 and 19 metabolites were significantly different in age groups and *APOE* genotype groups, respectively (Supplemental Fig. S10 and Supplemental Table S5). Four clusters of all significant metabolites had distinct correlations. Cluster A, comprising 5 metabolites (lactate, pyruvate, fumarate, hypoxanthine, and uracil), had inverse direct correlations with *APOE4*-TR old mice and had strong direct correlations with 3 other groups. However, cluster B and cluster C metabolites were associated with age. Ten metabolites in cluster B (methylamine, acetate, butyrate, propionate, arabinose, xylose, succinate, glucose, AMP, GTP) were more abundant in young mice, especially in *APOE3*-TR young compared with old mice. Fourteen metabolites in cluster C (asparagine, alanine, tryptophan, threonine, tyrosine, lysine, phenylalanine, glutamate, histidine, leucine, glutamine, valine, isoleucine, methionine) showed an opposite trend. Cluster D metabolites were divided into 2 subclusters: cluster D1, comprising 4 metabolites (2-oxoisocaproate, α-ketoisovalerate, 3-methyl-2-oxovalerate, urocanate), had direct correlations with *APOE4*-TR young mice; cluster D2, including 14 metabolites (isobutyrate, 1,3-dihydroxyacetone, lactaldehyde, aspartate, ornithine, ribose, xanthine, choline, glycine, creatine, taurine, 2-methylbutyric acid, ethanol, and formate), had positive correlations with old mice (**Fig. 3*A***). In addition, metabolite set enrichment analysis was used to identify significantly enriched pathways in metabolomics data associated with *APOE* genotype and age. Of the top 50 assigned pathways, the significant pathways in *APOE* genotype were ammonia recycling, urea cycle, and alanine metabolism (Supplemental Fig. S11 and Supplemental Table S6), whereas the significant pathways in age were ammonia recycling, urea cycle, glycine and serine metabolism, glutamate metabolism, and alanine metabolism (Supplemental Fig. S11*B* and Supplemental Table S7).

COIA was carried to explore the correlation between the composition of microbiota at OTU level and the fecal metabolome (Fig. 3*B*). The Monte Carlo permutation test revealed a high overall similarity in the structure between the 2 data sets, which was statistically significant (*RV* coefficient = 0.685; *P* = 0.01). The first 4 axes represented 72.5, 8.9, 7.0, and 2.6% of the explained variance, respectively, and so the analysis focused on the first axis. Each sample is represented by an arrow, where length of arrow indicates the divergence between 2 data sets. We observed that the aggregate arrow length was shorter in *APOE4* mice compared with that in *APOE3* mice, which indicated a higher consensus between microbiota composition and metabolites of *APOE4* mice compared with *APOE3* mice. The metabolites and OTUs that strongly correlated in the COIA axes were plotted on the first 2 COIA axes (Supplemental Fig. S12). Metabolites and bacterial OTUs were projected onto the same direction as samples, indicating that they were more abundant in those samples. There was an agreement between the metabolite abundance and the specific taxon abundance. Notably, SCFAs, including acetate, butyrate, and propionate, were located in the direction of butyrate-producing bacteria from *Clostridium* cluster IV genus and the families *Ruminococcaceae* and *Lachnospiraceae*.

## DISCUSSION

Although several recent studies have implicated a link between the gut microbiome, SCFAs, and the development of AD (3, 53–57), there is no direct study that establishes a link between gut microbiota composition and the strongest genetic risk factor for AD, *APOE* genotype. The current study marks the first analysis that compares gut microbiota composition in humans and transgenic mice with different *APOE* genotypes. Analysis of 16S rRNA gene sequences and fecal metabolome showed that *APOE* genotype correlated with abundance differences of several gut bacterial taxa, which may drive the difference in amino acids and SCFAs levels.

Higher levels of *Prevotellaceae* were evident in *APOE3/E3* carriers relative to other genotype subgroups, whereas higher levels of *Ruminococcaceae* were correlated with the *APOE2/E3* genotype (Fig. 1 and Supplemental Table S3) relative to *APOE4* carriers. Interestingly, loss of these bacteria has been reported to negatively correlate with neurodegenerative disorders and were noted as being less abundant in patients with Parkinson’s disease (58) and AD (3). A reduction of *Prevotellaceae* influenced mucin synthesis and increased mucosal permeability, allowing local and systemic exposure to bacterial endotoxin, which may lead to the accumulation of α-synuclein in the colon (59, 60). Aggregation-prone proteins such as β-amyloid and α-synuclein can propagate from the gut to the brain *via* the vagus nerve (61) and contribute to the pathogenesis of Parkinson’s disease, AD, and other neurodegenerative disorders (62–65). *Ruminococcaceae* are involved in the production of SCFAs, such that their depletion is causally linked to inflammation (66–68). These findings suggest that these bacteria might contribute to the protective effects of *APOE2* and *APOE3* alleles against AD relative to the *APOE4* genotype (10, 69, 70).

The high abundance of *Ruminococcaceae* in subjects of the *APOE2/E3* genotype was reflected by *Gemmiger* at the genus level. *Gemmiger* are strictly anaerobic bacteria that ferment a variety of carbohydrates to produce formic and *N*-butyric acids, often with small amounts of acetic, lactic, succinic, malonic, and pyruvic acids (71). In addition, we observed differences between *APOE* genotypes in *Clostridium* cluster IV, *Roseburia*, and *Clostridium* cluster XIVa, which are able to convert dietary fibers to SCFAs (72, 73). Although the butyrate-producing bacteria *Clostridium* cluster IV were modestly less abundant in human *APOE3/E3* individuals, they could be substituted by *Roseburia* with an increased abundance of these bacteria in the *APOE*3/*E3*. A slight increase of *Clostridium* cluster XIVa from *Lachnospiraceae* was seen in the human *APOE4/E4* genotype, which is consistent with the murine data. However, the increase of this genus in *APOE4/E4* may not substitute for the reduction of other butyrate-producing bacteria. Additionally, several genera, which were not correlated with *APOE* genotype in human gut microbiomes, were significantly different between *APOE3* and *APOE4* genotypes in murine gut microbiomes. These taxa could not be detected in human data because of: *1*) absence of some mouse gut microbiota in humans, such as *Mucispirillum*, *2*) differences in relative abundance of each individual taxa, and *3*) complexity in interactions of human gut microbiota with genetics, diet, and other environmental factors.

Incorporating the gut microbiota data with the corresponding metabolites in fecal water resulted in fecal samples across the *APOE* genotype being discriminated based on their metabolomic profiles. The lower concentrations of several fatty acids, especially SCFAs and their precursors (lactate and succinate), that were detected in *APOE4* old mice could be due to loss of butyrate-producing bacteria, similar to what we observed in humans with an *APOE4/E4* genotype (Fig. 1 and Supplemental Table S3). Although lower levels of SCFAs were noted in old *APOE3* mice, the lactate level was higher in this group, which is able to convert to butyrate by a subset of *Lachnospiraceae*, including *Eubacterium hallii* and *Anaerostipes caccae* (74). Similarly, *Bacteroides*, which were significantly more abundant in *APOE3* mice, have been reported to have propionate-producing capacity through the succinate pathway (75). Several SCFAs have been shown to inhibit the formation of toxic soluble β-amyloid aggregates *in vitro* (76) and consequently decrease the risk of AD. Interestingly, cosegregation of the fecal metabolomic profiles and the gut microbiome profiles as revealed by COIA suggests that the differences in gut microbiota associated with *APOE* genotype and age in *APOE*-TR mice are reflected in the segregation of metabolites, which may be clinically relevant. Specifically, SCFA level and the relative abundance of certain species from *Clostridium* cluster IV, *Ruminococcaceae*, and *Lachnospiraceae* showed a positive correlation. Future studies should further investigate strain level differences between *APOE* genotypes, the metabolic capacity of the microbiome, and the metabolomic profiles of fecal water extracts.

The collective evidence here suggests a link between *APOE* genotypes and gut microbiome composition. Loss of butyrate-producing bacteria and SCFAs in *APOE4* carriers might drive the impact of the *APOE4* allele on neuropathology. Our findings suggest a possible role of gut microbiota butyrate-producing bacteria as an intervention point to mitigate the impact of *APOE* genotype in the development of AD.

## Supplementary Material

This article includes supplemental data. Please visit *http://www.fasebj.org* to obtain this information.

## Abbreviations

AD: Alzheimer’s disease
APOE: apolipoprotein E
BMI: body mass index
CANN: Cognitive Ageing, Nutrition, and Neurogenesis
COB: chocolate, orange juice, and blackberry
COIA: coinertia analysis
LBP: LPS binding protein
OTU: operational taxonomic unit
PCoA: principal coordinates analysis
PERMANOVA: permutational multivariate ANOVA
QIIME: Quantitative Insights into Microbial Ecology
SCFA: short-chain fatty acid
TR: targeted replacement
