# Supplementary material for: APOE genotype influences the gut microbiome structure and function in humans and mice: relevance for Alzheimer’s disease pathophysiology
Source: FASEB J. 2019 Apr 8;33(7):8221–31. doi: 10.1096/fj.201900071R (PMC6593891; doi:10.1096/fj.201900071R)
Supplement: Supplementary file 1 [file fj.201900071R.sf1.pdf]

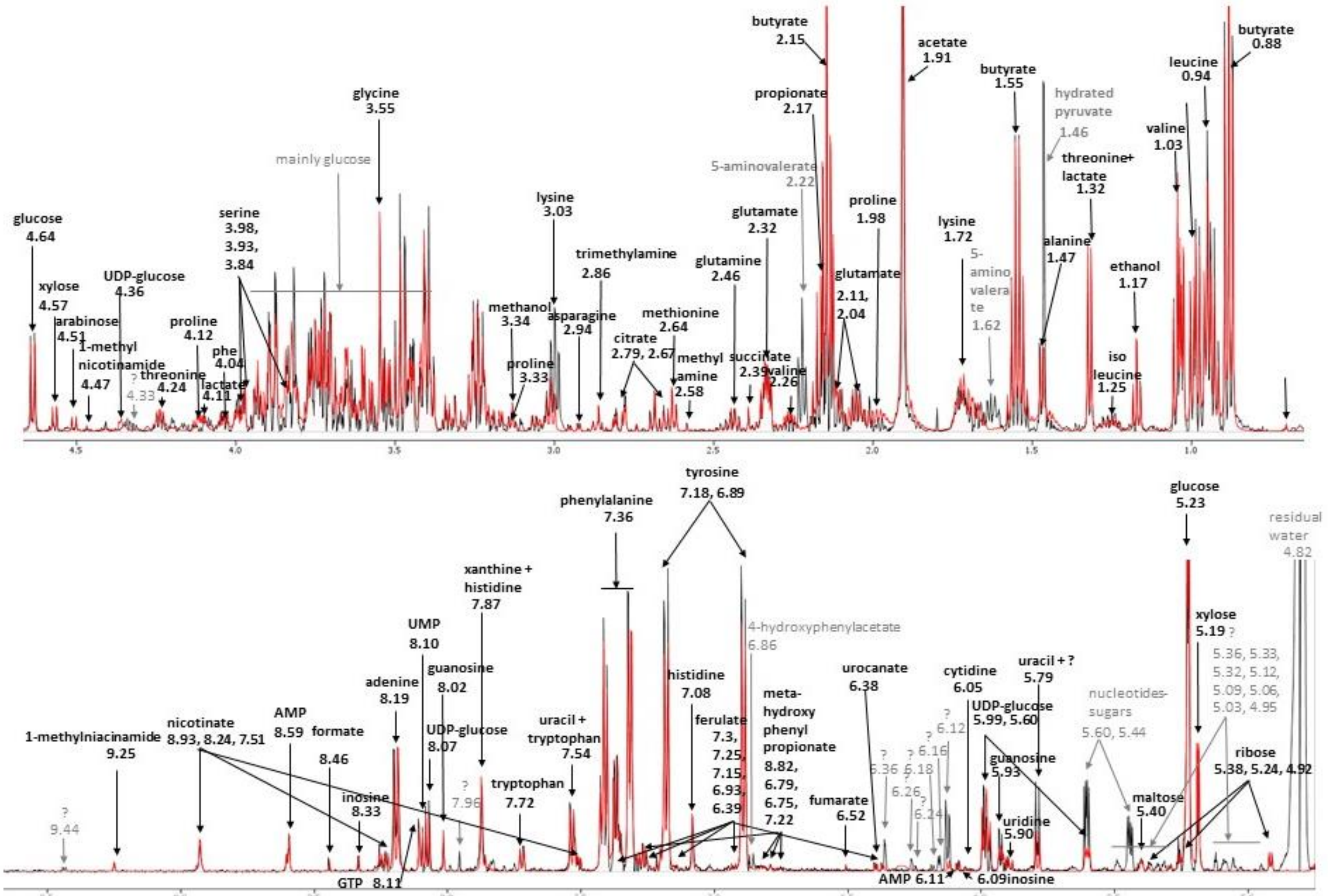

Figure S1. ^{1}H NMR spectra of faecal extract obtained from a murine sample at 600 MHz. The resonance signals were assigned to 63 metabolites which were identified using information found in the literature or on Human Metabolome Database website and by use of the 2D-NMR methods.
