# Supplementary material for: APOE genotype influences the gut microbiome structure and function in humans and mice: relevance for Alzheimer’s disease pathophysiology
Source: FASEB J. 2019 Apr 8;33(7):8221–31. doi: 10.1096/fj.201900071R (PMC6593891; doi:10.1096/fj.201900071R)
Supplement: Supplementary file 2 [file fj.201900071R.sf2.pdf]

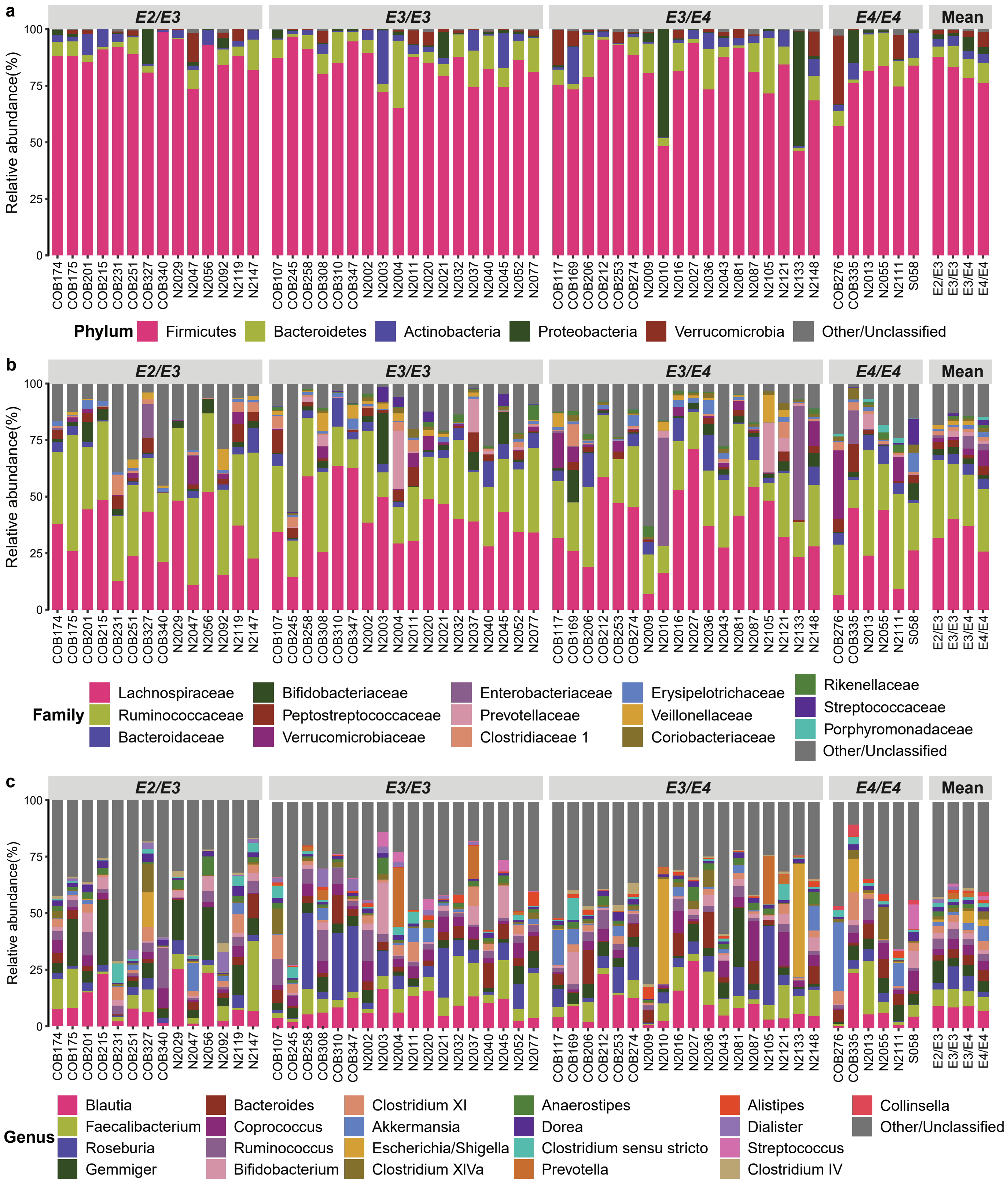

Figure S2. Relative abundance of human faecal microbiota taxa at (**a**) Phylum level, (**b**) Family level and (**c**) Genus level in subjects grouped according to *APOE* genotypes. Gut microbiota composition at phylum, family and genus levels exhibits different patterns according to human *APOE* genotype.
