# Supplementary material for: APOE genotype influences the gut microbiome structure and function in humans and mice: relevance for Alzheimer’s disease pathophysiology
Source: FASEB J. 2019 Apr 8;33(7):8221–31. doi: 10.1096/fj.201900071R (PMC6593891; doi:10.1096/fj.201900071R)
Supplement: Supplementary file 3 [file fj.201900071R.sf3.pdf]

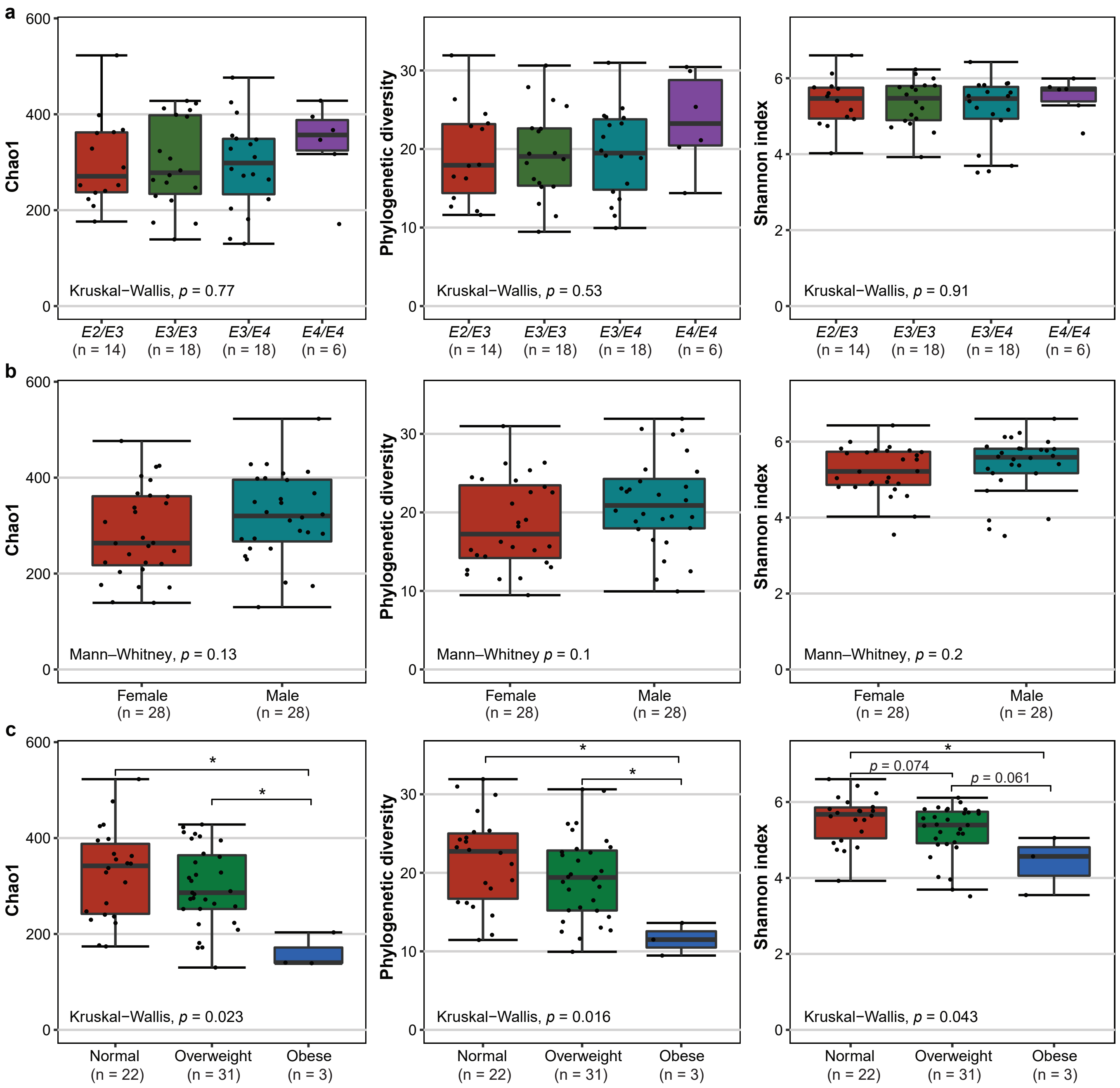

Figure S3. Boxplot of bacterial alpha-diversity indices for the microbiota from human faecal samples binned according to (**a**) *APOE* genotypes (**b**) sex or (**c**) BMI categories. No significant difference in alpha diversity was found between *APOE* genotypes or between males and females, whereas the microbiota alpha diversity of obese subjects was significantly lower than that of normal weight or overweight subjects. *P*-values were calculated by Mann–Whitney U test or Kruskal-Wallis H test followed by Dunn's multiple comparisons adjusting false discovery rate using the Benjamini-Hochberg correction. *$p < 0.05$.
