# Supplementary material for: APOE genotype influences the gut microbiome structure and function in humans and mice: relevance for Alzheimer’s disease pathophysiology
Source: FASEB J. 2019 Apr 8;33(7):8221–31. doi: 10.1096/fj.201900071R (PMC6593891; doi:10.1096/fj.201900071R)
Supplement: Supplementary file 4 [file fj.201900071R.sf4.pdf]

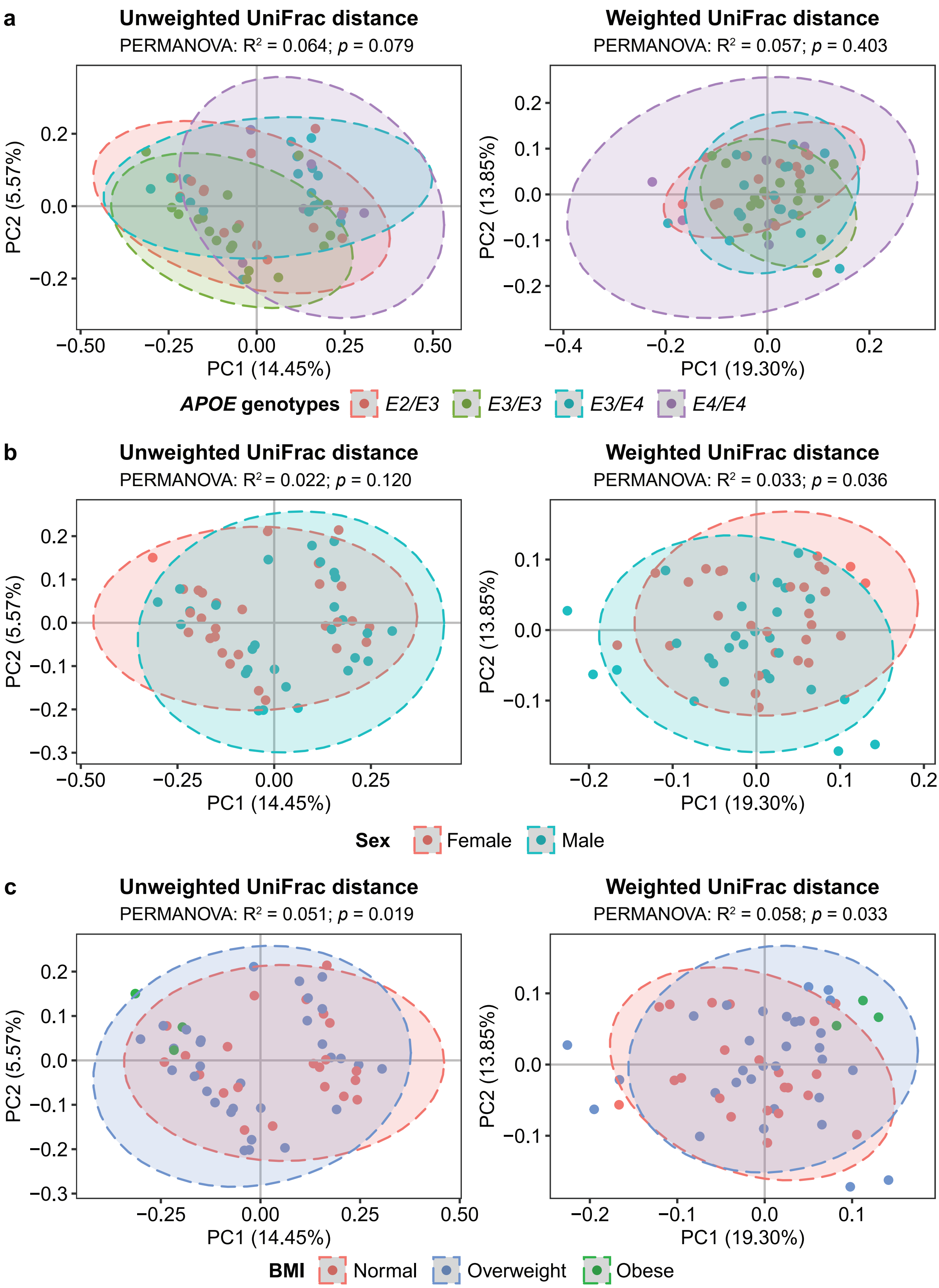

Figure S4. Principle Coordinates Analysis based on unweighted and weighted UniFrac distances of bacterial 16S rRNA amplicon sequences from human faecal samples showing gut microbiota beta diversity grouped according to (**a**) *APOE* genotypes, (**b**) sex or (**c**) BMI categories. Beta diversity analysis reveals revealed significant separation of microbial communities according to gender and BMI categories, but not *APOE* genotypes. The significant differences between groups were determined by PERMANOVA tests.
