# Supplementary material for: APOE genotype influences the gut microbiome structure and function in humans and mice: relevance for Alzheimer’s disease pathophysiology
Source: FASEB J. 2019 Apr 8;33(7):8221–31. doi: 10.1096/fj.201900071R (PMC6593891; doi:10.1096/fj.201900071R)
Supplement: Supplementary file 5 [file fj.201900071R.sf5.pdf]

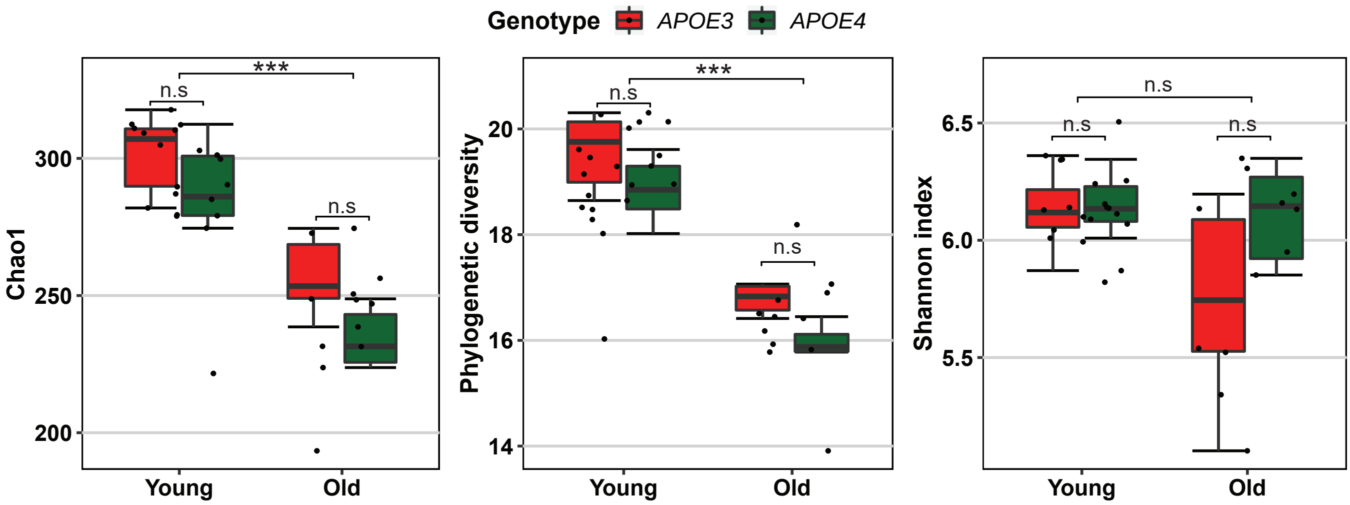

Figure S5. Boxplot of bacterial alpha-diversity indices for the faecal microbiota from mice grouped according to *APOE* genotype and age. No significant difference in alpha diversity between *APOE*3 and *APOE*4 genotypes was observed in transgenic mice, but both chao1 and phylogenetic diversity were much higher in young mice compared to old mice. *P*-value was calculated by Mann–Whitney U test for two *APOE* genotypes or two age groups. ***$p < 0.001$; n.s, not significant.
