# Supplementary material for: APOE genotype influences the gut microbiome structure and function in humans and mice: relevance for Alzheimer’s disease pathophysiology
Source: FASEB J. 2019 Apr 8;33(7):8221–31. doi: 10.1096/fj.201900071R (PMC6593891; doi:10.1096/fj.201900071R)
Supplement: Supplementary file 7 [file fj.201900071R.sf7.pdf]

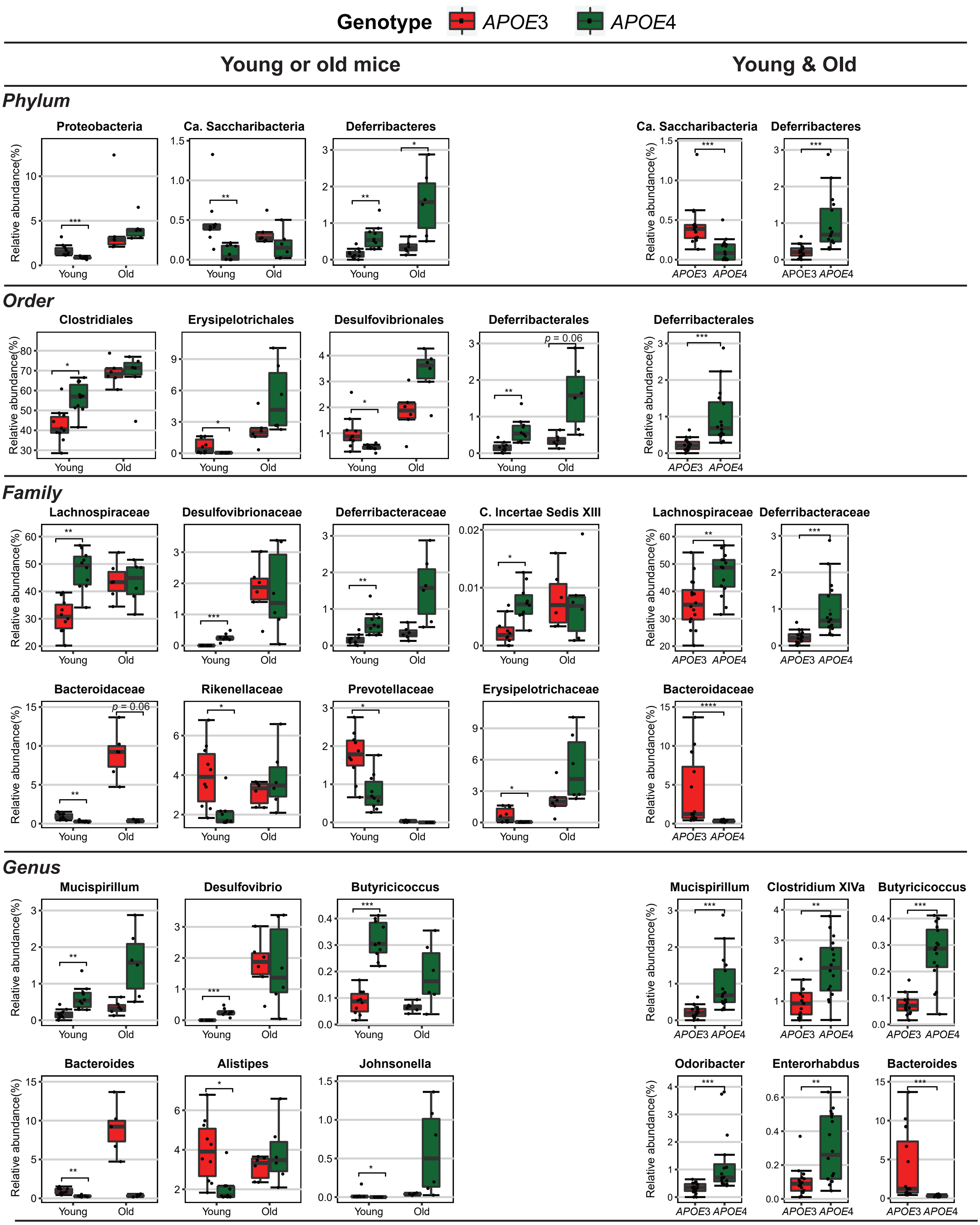

Figure S7. Box plot of the relative abundance of significantly differentially abundant faecal microbiota taxa from phylum to genus associated with murine *APOE* genotypes in young mice samples, old mice samples and both age groups combined. Statistical significances between *APOE*3 and *APOE*4 were determined by the Mann–Whitney U test and were corrected for the multiple comparison using the Benjamini–Hochberg adjustment, *$p < 0.05$, **$p < 0.01$, ***$p < 0.001$.
