# Supplementary material for: APOE genotype influences the gut microbiome structure and function in humans and mice: relevance for Alzheimer’s disease pathophysiology
Source: FASEB J. 2019 Apr 8;33(7):8221–31. doi: 10.1096/fj.201900071R (PMC6593891; doi:10.1096/fj.201900071R)
Supplement: Supplementary file 8 [file fj.201900071R.sf8.pdf]

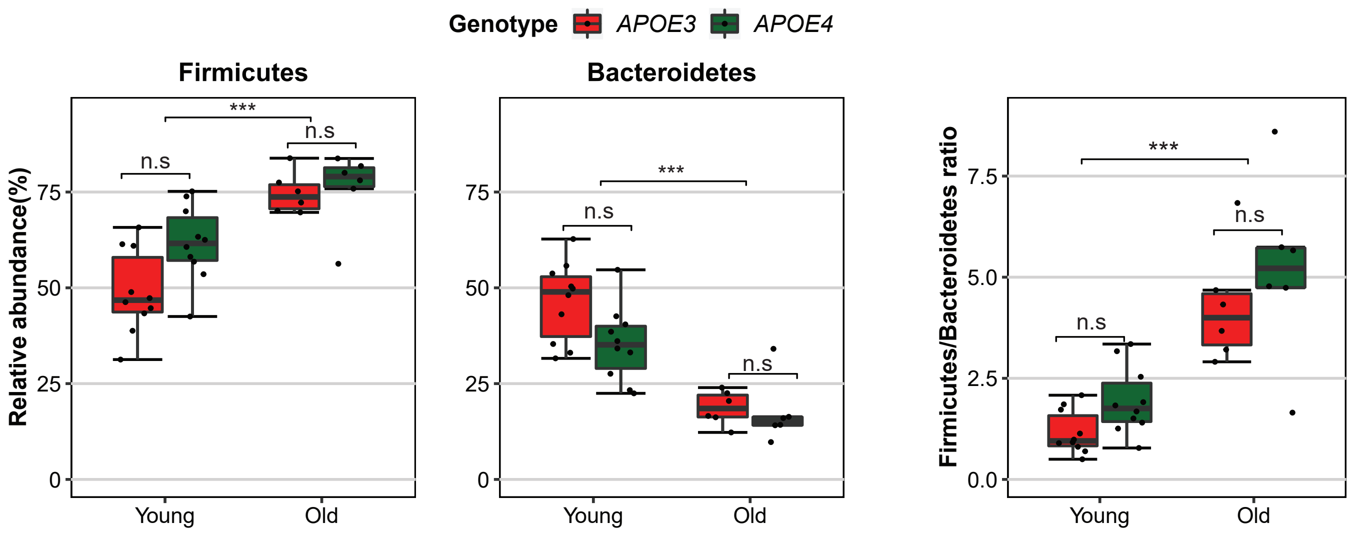

Figure S8. Box plot of the relative abundance from murine faecal samples of Firmicutes, Bacteroidetes and Firmicutes/Bacteroidetes ratio according to age and *APOE* genotypes. The Firmicutes/Bacteroidetes ratio was higher in old mice than young mice. *P*-values were calculated by Mann–Whitney U test for testing the significant difference between *APOE* genotype groups or age groups, ***$p < 0.001$; n.s, not significant.
