# Supplementary material for: APOE genotype influences the gut microbiome structure and function in humans and mice: relevance for Alzheimer’s disease pathophysiology
Source: FASEB J. 2019 Apr 8;33(7):8221–31. doi: 10.1096/fj.201900071R (PMC6593891; doi:10.1096/fj.201900071R)
Supplement: Supplementary file 9 [file fj.201900071R.sf9.pdf]

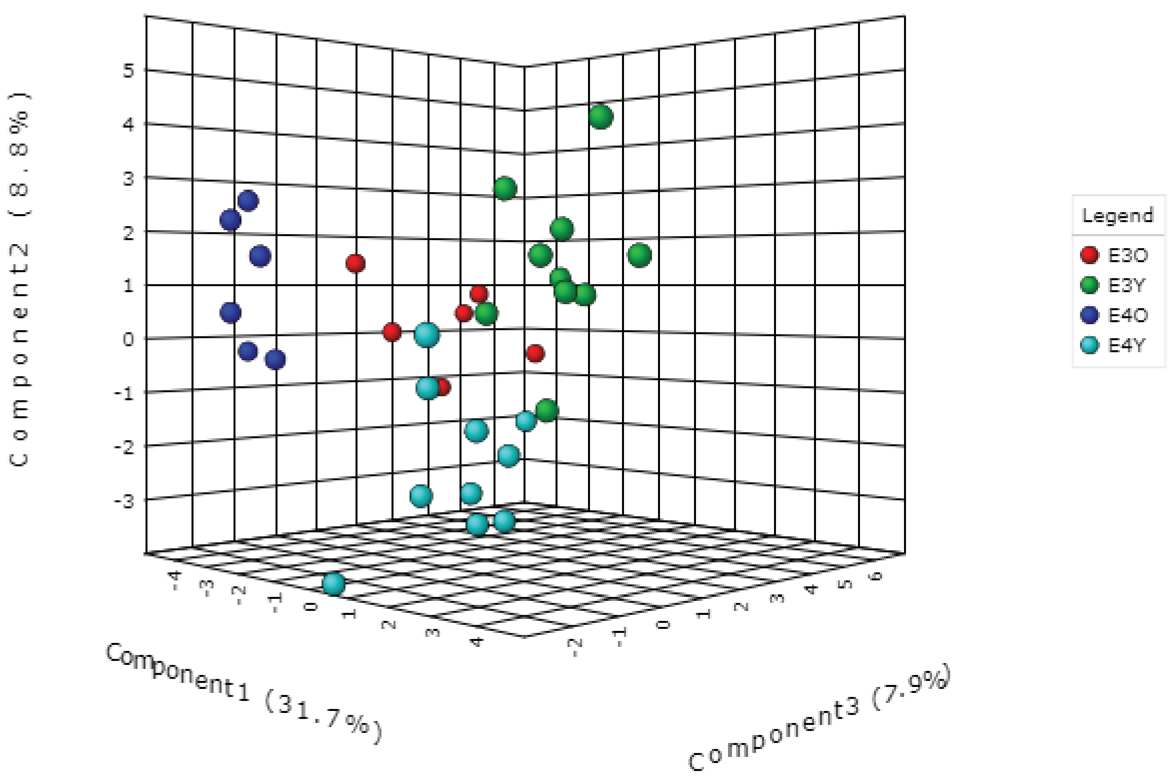

Figure S9. Sparse PLS Discriminant Analysis (sPLS-DA) shows a trend for microbiota separation according to age and *APOE* genotype. E3Y, *APOE3* young mice; E4Y, *APOE4* young mice; E3O, *APOE3* old mice; E4O, *APOE4* old mice.
