# Supplementary material for: APOE genotype influences the gut microbiome structure and function in humans and mice: relevance for Alzheimer’s disease pathophysiology
Source: FASEB J. 2019 Apr 8;33(7):8221–31. doi: 10.1096/fj.201900071R (PMC6593891; doi:10.1096/fj.201900071R)
Supplement: Supplementary file 10 [file fj.201900071R.sf10.pdf]

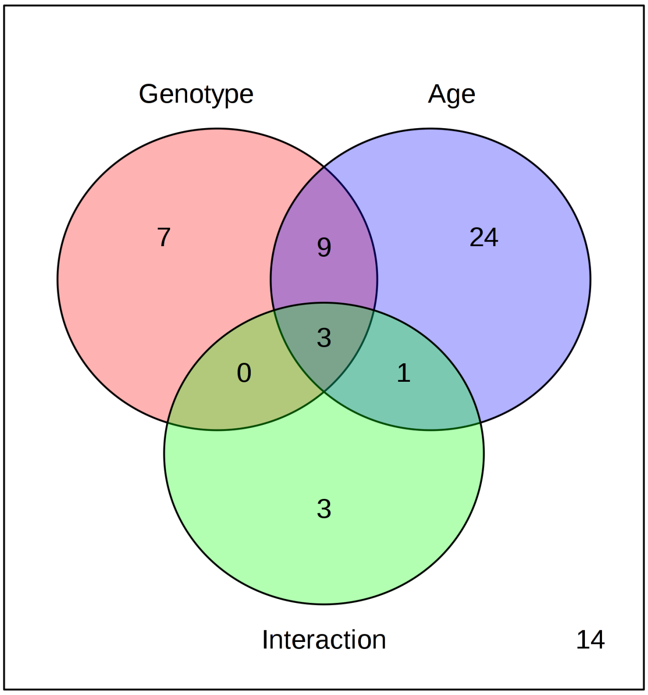

Figure S10. Venn diagram summary of statistically significant metabolites according to *APOE* genotype and age were conducted by two-way ANOVA with False Discovery Rate (FDR) correction. A total of 39 and 19 metabolites were significantly different in age groups and *APOE g*enotype groups respectively.
