# Supplementary material for: APOE genotype influences the gut microbiome structure and function in humans and mice: relevance for Alzheimer’s disease pathophysiology
Source: FASEB J. 2019 Apr 8;33(7):8221–31. doi: 10.1096/fj.201900071R (PMC6593891; doi:10.1096/fj.201900071R)
Supplement: Supplementary file 11 [file fj.201900071R.sf11.pdf]

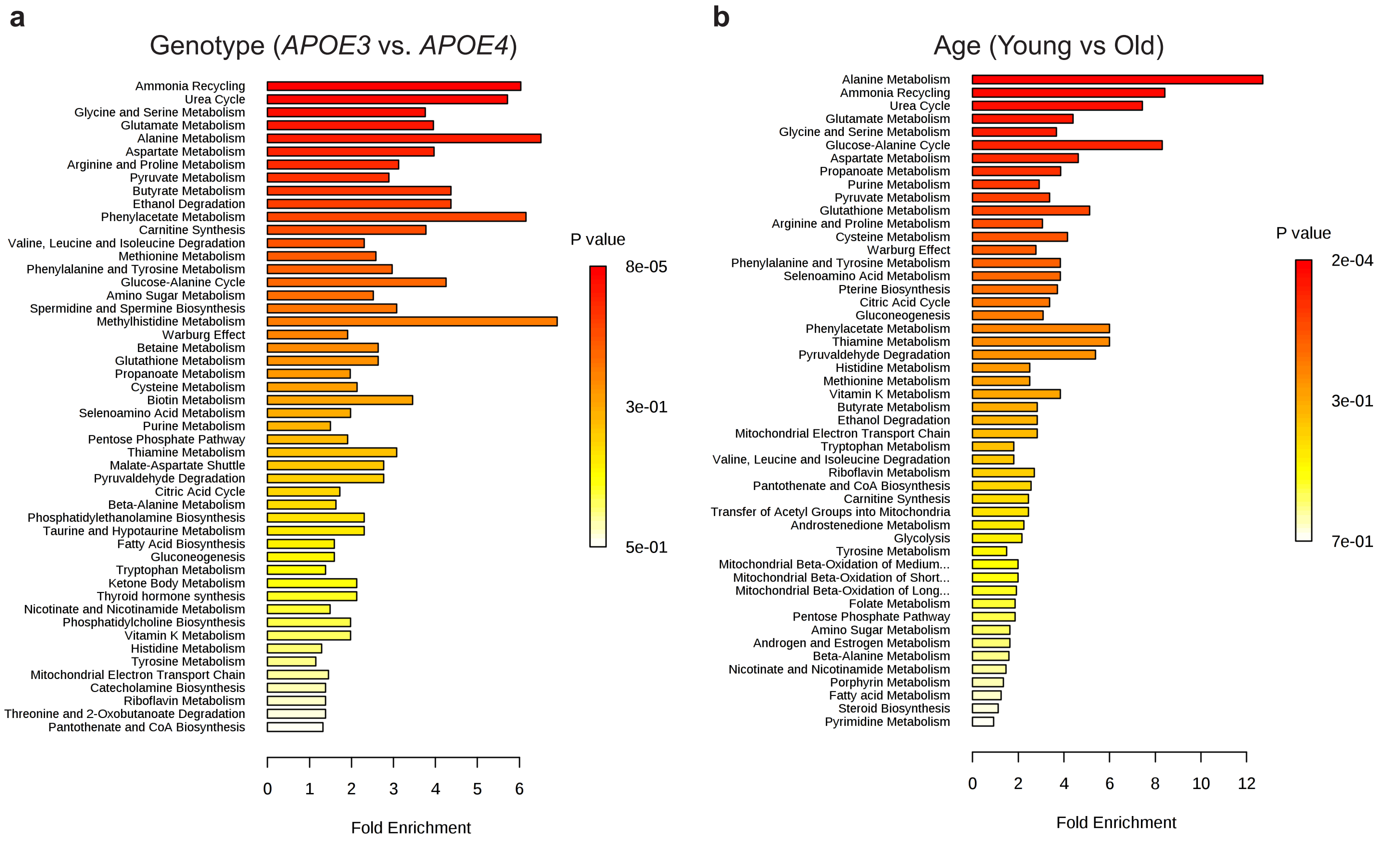

Figure S11. Metabolites Set Enrichment associated to (**a**) *APOE* genotype and (**b**) age for faecal metabolites. The significant pathways in *APOE* genotype were ammonia recycling, urea cycle, and alanine metabolism, while the significant pathways in age were ammonia recycling, urea cycle, glycine and serine metabolism, glutamate metabolism and alanine metabolism.
