# Supplementary material for: APOE genotype influences the gut microbiome structure and function in humans and mice: relevance for Alzheimer’s disease pathophysiology
Source: FASEB J. 2019 Apr 8;33(7):8221–31. doi: 10.1096/fj.201900071R (PMC6593891; doi:10.1096/fj.201900071R)
Supplement: Supplementary file 13 [file fj.201900071R.st1.pdf]

Table S1. Descriptive statistics of 56 study participants.

APOE genotypes	Sample names	Age	Sex	BMI	BMI category	Haptoglobin (mg/ml)	LBP (μg/ml)
E2/E3	COB174	64	M	24.9	Normal	0.15	2.4
	COB175	70	M	27.2	Overweight	0.4	1.8
	COB201	68	M	26.2	Overweight	0.38	1.7
	COB215	63	F	21.5	Normal	0.61	1.6
	COB231	74	F	21.9	Normal	0.11	1.5
	COB251	77	F	25.0	Normal	0.95	1.9
	COB327	64	M	26.6	Overweight	0.68	1.9
	COB340	61	F	28.6	Overweight	1.47	1.9
	N2029	72	F	23.2	Normal	0.48	2.6
	N2047	73	F	26.0	Overweight	0.95	3.5
	N2056	70	F	25.1	Overweight	0.46	5.1
	N2092	70	M	22.5	Normal	0.5	2.4
	N2119	68	M	27.4	Overweight	0.86	4.1
	N2147	66	M	24.0	Normal	0.53	2.6
E3/E3	COB107	71	M	27.5	Overweight	1.16	2.7
	COB245	72	M	24.1	Normal	1.25	1.8
	COB258	67	F	24.8	Normal	0.15	1.7
	COB308	75	M	25.6	Overweight	0.6	1.6
	COB310	70	F	25.7	Overweight	0.15	1.6
	COB347	65	F	25.7	Overweight	1.09	9.5
	N2002	66	F	23.6	Normal	0.2	2
	N2003	70	F	32.1	Obese	0.11	2.4
	N2004	69	M	28.4	Overweight	0.66	1.7
	N2011	67	F	29.2	Overweight	1.05	2
	N2020	76	M	27.2	Overweight	0.85	3.2
	N2021	65	F	28.4	Overweight	0.15	2.3
	N2032	78	M	28.2	Overweight	0.28	2.7
	N2037	56	M	20.7	Normal	0.66	2.6
	N2040	66	M	23.8	Normal	0.82	2.9
	N2045	67	F	25.0	Overweight	0.33	2.7
	N2052	69	M	27.2	Overweight	0.08	5.2
	N2077	64	F	26.9	Overweight	0.81	2.7
E3/E4	COB117	74	M	26.2	Overweight	1.74	1.8
	COB169	67	F	24.8	Normal	1.36	1.7
	COB206	72	M	28.7	Overweight	0.03	1.2
	COB212	67	M	26.0	Overweight	2.02	1.8
	COB253	68	F	22.5	Normal	0.64	1.6
	COB274	67	F	28.7	Overweight	1.25	1.3
	N2009	76	M	26.2	Overweight	0.64	3.1
	N2010	68	F	31.3	Obese	1.37	2.4
	N2016	66	F	20.4	Normal	0.26	4.1
	N2027	68	F	32.0	Obese	0.45	2.4
	N2036	71	M	26.1	Overweight	0.57	2.5
	N2043	68	F	23.7	Normal	0.37	2.3
	N2081	67	F	27.7	Overweight	0.22	2
	N2087	68	F	23.3	Normal	0.02	2.8
	N2105	63	M	29.2	Overweight	0.08	4.1
	N2121	67	M	24.6	Normal	0.25	2

APOE genotypes	Sample names	Age	Sex	BMI	BMI category	Haptoglobin (mg/ml)	LBP (µg/ml)
	N2133	69	M	26.4	Overweight	0.11	2
	N2148	69	M	22.7	Normal	0.39	2.4
**E4/E4**	COB276	72	M	26.9	Overweight	0.04	1.9
	COB335	67	F	25.6	Overweight	1.52	2.5
	N2013	59	F	23.4	Normal	0.88	2
	N2055	62	F	22.6	Normal	0.43	3.1
	N2111	72	M	24.1	Normal	0.29	4.9
	S058	74	M	27.2	Overweight	-	-
