# Supplementary material for: APOE genotype influences the gut microbiome structure and function in humans and mice: relevance for Alzheimer’s disease pathophysiology
Source: FASEB J. 2019 Apr 8;33(7):8221–31. doi: 10.1096/fj.201900071R (PMC6593891; doi:10.1096/fj.201900071R)
Supplement: Supplementary file 14 [file fj.201900071R.st2.pdf]

Table S2. PERMANOVA tests of clinical parameters with the unweighted and weighted UniFrac distance matrices.

**Parameters**	**Unweighted UniFrac**		**Weighted UniFrac**	
	**R^2**	***p***	**R^2**	***p***
APOE genotypes	0.064	0.079	0.057	0.403
Sex	0.022	0.120	0.033	0.036
BMI categories	0.051	0.019	0.058	0.033
Haptoglobin	0.016	0.650	0.017	0.410
LBP	0.016	0.720	0.013	0.700
