# Supplementary material for: APOE genotype influences the gut microbiome structure and function in humans and mice: relevance for Alzheimer’s disease pathophysiology
Source: FASEB J. 2019 Apr 8;33(7):8221–31. doi: 10.1096/fj.201900071R (PMC6593891; doi:10.1096/fj.201900071R)
Supplement: Supplementary file 15 [file fj.201900071R.st3.pdf]

Table S3. Taxa associated with human *APOE* genotype detected by Kruskal-Wallis test with Dunn's multiple comparision correction

Taxa		Kruskal-Wallis test	Dunn's multiple comparison with BH adjustment						Means			
			E2/E3 - E3/E3	E2/E3 - E3/E4	E3/E3 - E3/E4	E2/E3 - E4/E4	E3/E3 - E4/E4	E3/E4 - E4/E4	E2/E3	E3/E3	E3/E4	E4/E4
Phylum	Firmicutes	0.041	0.115	0.029	0.248	0.039	0.126	0.211	87.90	83.42	78.59	76.20
Class	Firmicutes.Clostridia	0.035	0.091	0.051	0.323	0.020	0.094	0.137	81.05	75.21	71.05	67.92
Order	Firmicutes.Clostridia.Clostridiales	0.036	0.091	0.050	0.319	0.021	0.098	0.143	80.90	75.08	70.91	67.74
Family	Bacteroidetes.Bacteroidia.Bacteroidales.Prevotellaceae	0.009	0.008	0.085	0.103	0.015	0.340	0.088	0.14	2.93	1.79	1.40
	Firmicutes.Clostridia.Clostridiales.Ruminococcaceae	0.004	0.004	0.002	0.383	0.072	0.348	0.352	34.36	24.39	22.71	27.45
Genus	Firmicutes.Clostridia.Clostridiales.Lachnospiraceae.Clostridium XlVa	0.094	0.140	0.486	0.162	0.044	0.177	0.078	1.56	1.70	1.20	3.69
	Firmicutes.Clostridia.Clostridiales.Lachnospiraceae.Roseburia	0.057	0.080	0.099	0.328	0.365	0.063	0.082	2.77	9.93	6.86	2.37
	Firmicutes.Clostridia.Clostridiales.Ruminococcaceae.Clostridium IV	0.043	0.027	0.480	0.039	0.371	0.232	0.281	1.10	0.59	1.13	0.73
	Firmicutes.Clostridia.Clostridiales.Ruminococcaceae.Gemmiger	0.086	0.050	0.086	0.476	0.400	0.184	0.228	10.14	3.71	4.64	4.96
