# Supplementary material for: APOE genotype influences the gut microbiome structure and function in humans and mice: relevance for Alzheimer’s disease pathophysiology
Source: FASEB J. 2019 Apr 8;33(7):8221–31. doi: 10.1096/fj.201900071R (PMC6593891; doi:10.1096/fj.201900071R)
Supplement: Supplementary file 16 [file fj.201900071R.st4.pdf]

Table S4. Results from Mann-Whitney U tests after Benjamini–Hochberg corrections for differences in relative abundance between *APOE* 3 and *APOE* 4 in young mice samples, old mice samples and both age groups.

Taxa		Young mice			Old mice			Young & Old mice		
		adj.p	mean (*APOE3*)	mean (*APOE4*)	adj.p	mean (*APOE3*)	mean (*APOE4*)	adj.p	mean (*APOE3*)	mean (*APOE4*)
Phylum	Candidatus Saccharibacteria	0.002	0.48	0.08	0.372	0.33	0.18	0.000	0.43	0.12
	Deferribacteres	0.001	0.16	0.61	0.030	0.35	1.57	0.000	0.23	0.97
	Proteobacteria	0.000	1.71	0.86	0.396	4.27	4.11	0.594	2.67	2.08
Class	Deferribacteres.Deferribacteres	0.003	0.16	0.61	0.061	0.35	1.57	0.000	0.23	0.97
	Firmicutes.Erysipelotrichia	0.029	0.67	0.04	0.312	2.18	5.28	1.000	1.23	2.01
	Proteobacteria.Deltaproteobacteria	0.023	1.03	0.45	0.452	1.84	3.34	1.000	1.33	1.53
Order	Deferribacteres.Deferribacteres.Deferribacterales	0.003	0.16	0.61	0.065	0.35	1.57	0.000	0.23	0.97
	Firmicutes.Clostridia.Clostridiales	0.020	42.39	56.34	1.000	68.93	67.65	1.000	52.34	60.58
	Firmicutes.Erysipelotrichia.Erysipelotrichales	0.032	0.67	0.04	0.338	2.18	5.28	1.000	1.23	2.01
	Proteobacteria.Deltaproteobacteria.Desulfovibrionales	0.025	1.03	0.45	0.494	1.84	3.34	1.000	1.33	1.53
Family	Bacteroidetes.Bacteroidia.Bacteroidales.Bacteroidaceae	0.001	0.89	0.28	0.058	8.96	0.35	0.000	3.92	0.31
	Bacteroidetes.Bacteroidia.Bacteroidales.Prevotellaceae	0.038	1.76	0.79	1.000	0.03	0.00	1.000	1.11	0.49
	Bacteroidetes.Bacteroidia.Bacteroidales.Rikenellaceae	0.015	3.98	2.01	1.000	3.10	3.84	0.319	3.65	2.70
	Deferribacteres.Deferribacteres.Deferribacterales.Deferribacteraceae	0.005	0.16	0.61	0.108	0.35	1.57	0.000	0.23	0.97
	Firmicutes.Clostridia.Clostridiales.Clostridiales Incertae Sedis XIII	0.048	0.00	0.01	1.000	0.01	0.01	0.909	0.00	0.01
	Firmicutes.Clostridia.Clostridiales.Lachnospiraceae	0.002	30.93	47.83	1.000	43.80	43.34	0.039	35.76	46.15
	Firmicutes.Erysipelotrichia.Erysipelotrichales.Erysipelotrichaceae	0.048	0.67	0.04	0.545	2.18	5.28	1.000	1.23	2.01
	Proteobacteria.Deltaproteobacteria.Desulfovibrionales.Desulfovibrionaceae	0.000	0.00	0.26	1.000	1.80	1.72	1.000	0.68	0.81
Genus	Actinobacteria.Actinobacteria.Coriobacteriales.Coriobacteriaceae.Enterorhabdus	0.057	0.10	0.40	1.000	0.10	0.13	0.043	0.10	0.30
	Bacteroidetes.Bacteroidia.Bacteroidales.Bacteroidaceae.Bacteroides	0.002	0.89	0.28	0.104	8.96	0.35	0.000	3.92	0.31
	Bacteroidetes.Bacteroidia.Bacteroidales.Porphyromonadaceae.Odoribacter	0.720	0.42	0.80	0.104	0.18	1.92	0.001	0.33	1.22
	Bacteroidetes.Bacteroidia.Bacteroidales.Rikenellaceae.Alistipes	0.028	3.98	2.01	1.000	3.10	3.84	0.577	3.65	2.70
	Deferribacteres.Deferribacteres.Deferribacterales.Deferribacteraceae.Mucispirillum	0.009	0.16	0.61	0.195	0.35	1.57	0.000	0.23	0.97
	Firmicutes.Clostridia.Clostridiales.Lachnospiraceae.Clostridium XlVa	0.171	0.97	1.94	1.000	1.05	2.33	0.032	1.00	2.09
	Firmicutes.Clostridia.Clostridiales.Lachnospiraceae.Johnsonella	0.011	0.02	0.00	1.000	0.04	0.60	1.000	0.03	0.22
	Firmicutes.Clostridia.Clostridiales.Ruminococcaceae.Butyricicoccus	0.001	0.08	0.32	1.000	0.07	0.19	0.000	0.08	0.27
	Proteobacteria.Deltaproteobacteria.Desulfovibrionales.Desulfovibrionaceae.Desulfovibrio	0.001	0.00	0.26	1.000	1.80	1.72	1.000	0.68	0.81
