# Supplementary material for: APOE genotype influences the gut microbiome structure and function in humans and mice: relevance for Alzheimer’s disease pathophysiology
Source: FASEB J. 2019 Apr 8;33(7):8221–31. doi: 10.1096/fj.201900071R (PMC6593891; doi:10.1096/fj.201900071R)
Supplement: Supplementary file 17 [file fj.201900071R.st5.pdf]

Table S5. List of metabolites differentially associated with *APOE* genotype and Age based on two-way ANOVA

Group	Compound ID	HMDB ID	KEGG ID	Post-hoc test following a Kruskal-Wallis test with BH adjustment			Means (mmol/Kg)			
				Genotype (adj.p)	Age (adj.p)	Interaction (adj.p)	E3Y	E4Y	E3O	E4O
Alcohols	1,3-Dihydroxyacetone	HMDB01882	C00184	0.678	0.008	0.796	0.02	0.03	0.04	0.07
Alcohols	Ethanol	HMDB00108	C00469	0.851	0.001	0.827	1.23	1.17	1.42	2.00
Alcohols	Lactaldehyde	HMDB03052	C00424	0.810	0.013	0.494	0.16	0.15	0.17	0.28
Amines	Choline	HMDB00097	C00114	0.851	0.033	0.607	0.66	0.53	0.73	0.73
Amines	Creatine	HMDB00064	C00300	0.810	0.000	0.940	0.58	0.52	0.78	1.05
Amines	Methylamine	HMDB00164	C00218	0.013	0.000	0.587	0.18	0.09	0.05	0.05
Amines	Taurine	HMDB00251	C00245	0.384	0.000	0.527	3.09	3.71	8.44	10.67
Aminoacids	Alanine	HMDB00161	C00041	0.013	0.000	0.866	1.97	2.65	3.11	5.55
Aminoacids	Asparagine	HMDB00168	C00152	0.046	0.001	0.453	0.64	0.72	0.94	2.03
Aminoacids	Aspartate	HMDB00191	C00049	0.245	0.002	0.153	0.59	0.54	0.53	1.09
Aminoacids	Glutamate	HMDB03339	C00217	0.790	0.000	0.932	6.35	5.77	7.63	9.19
Aminoacids	Glutamine	HMDB00641	C00064	0.103	0.000	0.388	1.23	1.44	2.17	2.66
Aminoacids	Glycine	HMDB00123	C00037	0.006	0.000	0.039	2.64	3.93	3.88	4.63
Aminoacids	Histidine	HMDB00177	C00135	0.164	0.000	0.609	0.58	0.63	0.79	0.99
Aminoacids	Isoleucine	HMDB00172	C00407	0.440	0.000	0.796	2.58	2.66	3.74	4.83
Aminoacids	Leucine	HMDB00687	C00123	0.100	0.000	0.345	3.81	4.53	5.93	7.22
Aminoacids	Lysine	HMDB00182	C00047	0.073	0.000	0.362	3.37	4.02	4.72	5.88
Aminoacids	Methionine	HMDB00696	C00073	0.223	0.000	0.575	1.73	1.87	2.08	2.62
Aminoacids	Ornithine	HMDB00214	C00077	0.714	0.028	0.274	0.11	0.07	0.15	0.25
Aminoacids	Phenylalanine	HMDB00159	C00079	0.103	0.001	0.362	1.99	2.29	2.41	2.95
Aminoacids	Threonine	HMDB00167	C00188	0.087	0.001	0.256	2.41	2.94	3.11	3.77
Aminoacids	Tryptophan	HMDB00929	C00078	0.156	0.000	0.918	0.35	0.39	0.50	0.66
Aminoacids	Tyrosine	HMDB00158	C00082	0.156	0.001	0.902	2.43	2.60	2.79	3.73
Aminoacids	Valine	HMDB00883	C00183	0.031	0.000	0.203	2.87	3.48	4.54	5.67
Carbohydrates	Arabinose	HMDB29942	C00259	0.047	0.000	0.608	2.44	1.87	0.47	0.35
Carbohydrates	Glucose	HMDB00122	C00031	0.618	0.507	0.039	20.88	12.09	8.54	18.18
Carbohydrates	Ribose	HMDB00283	C00121	0.768	0.027	0.199	3.06	3.52	3.80	4.14
Carbohydrates	Xylose	HMDB00098	C00181	0.019	0.000	0.609	4.41	2.91	0.87	0.63

Group	Compound ID	HMDB ID	KEGG ID	Post-hoc test following a Kruskal-Wallis test with BH adjustment			Means (mmol/Kg)			
				Genotype (adj.p)	Age (adj.p)	Interaction (adj.p)	E3Y	E4Y	E3O	E4O
Fatty acids	2-methylbutyric acid	HMDB0033742	C15492	0.714	0.033	0.931	0.11	0.15	0.41	0.38
Fatty acids	2-Oxoisocaproate	HMDB00695	C00233	0.223	0.003	0.041	0.42	0.69	0.29	0.27
Fatty acids	3-Methyl-2-oxovalerate	HMDB00491	C03465	0.044	0.579	0.153	0.34	0.69	0.32	0.35
Fatty acids	Acetate	HMDB00042	C00033	0.073	0.000	0.954	39.45	30.70	15.13	15.30
Fatty acids	Alpha-ketoisovaleric acid	HMDB00019	C00141	0.103	0.077	0.034	0.28	0.52	0.25	0.23
Fatty acids	Butyrate	HMDB00039	C00246	0.509	0.000	0.153	15.44	9.70	3.02	4.87
Fatty acids	Formate	HMDB00142	C00058	0.031	0.001	0.902	0.25	0.16	0.35	0.29
Fatty acids	Fumarate	HMDB00134	C00122	0.049	0.302	0.840	0.08	0.05	0.05	0.05
Fatty acids	Isobutyrate	HMDB01873	C02632	0.046	0.836	0.796	0.47	0.33	0.45	0.29
Fatty acids	Lactate	HMDB00190	C00186	0.000	0.000	0.000	2.22	1.81	2.49	0.00
Fatty acids	Propionate	HMDB00237	C00163	0.006	0.000	0.735	6.19	3.55	2.70	1.82
Fatty acids	Pyruvate	HMDB00243	C00022	0.044	0.018	0.056	5.44	4.85	4.30	3.09
Fatty acids	Succinate	HMDB00254	C00042	0.826	0.001	0.494	1.20	0.96	0.35	0.58
Fatty acids	Urocanate	HMDB00301	C00785	0.006	0.254	0.165	0.09	0.17	0.11	0.15
Pirimidines	Uracil	HMDB00300	C00106	0.011	0.339	0.165	1.99	1.63	1.79	1.23
Purine	AMP	HMDB00045	C00020	0.006	0.002	0.002	0.15	0.04	0.03	0.05
Purine	GTP	HMDB12282	C00404	0.006	0.168	0.274	0.32	0.15	0.17	0.17
Purine	Hypoxanthine	HMDB00157	C00262	0.006	0.219	0.587	1.76	1.36	1.35	1.21
Purine	Xanthine	HMDB00292	C00385	0.151	0.059	0.023	1.20	1.22	1.74	1.08

Note. E3Y, APOE3 young mice; E4Y, APOE4 young mice; E3O, APOE3 old mice; E4O, APOE4 old mice.
