# Supplementary material for: APOE genotype influences the gut microbiome structure and function in humans and mice: relevance for Alzheimer’s disease pathophysiology
Source: FASEB J. 2019 Apr 8;33(7):8221–31. doi: 10.1096/fj.201900071R (PMC6593891; doi:10.1096/fj.201900071R)
Supplement: Supplementary file 18 [file fj.201900071R.st6.pdf]

Table S6. Metabolites Set Enrichment associated to *APOE* genotype for the faecal metabolites (top 50 pathways).

Pathways	Total	Expected	Hits	Raw p	Holm p	FDR
Alanine Metabolism	17	0.315	4	0.000174	0.017	0.00903
Ammonia Recycling	32	0.594	5	0.000184	0.0179	0.00903
Urea Cycle	29	0.538	4	0.0015	0.144	0.0491
Glutamate Metabolism	49	0.909	4	0.0106	1	0.258
Glycine and Serine Metabolism	59	1.09	4	0.0201	1	0.341
Glucose-Alanine Cycle	13	0.241	2	0.0225	1	0.341
Aspartate Metabolism	35	0.649	3	0.0244	1	0.341
Propanoate Metabolism	42	0.779	3	0.0393	1	0.462
Purine Metabolism	74	1.37	4	0.0424	1	0.462
Pyruvate Metabolism	48	0.891	3	0.0552	1	0.495
Glutathione Metabolism	21	0.39	2	0.0555	1	0.495
Arginine and Proline Metabolism	53	0.983	3	0.0705	1	0.567
Cysteine Metabolism	26	0.482	2	0.0814	1	0.567
Warburg Effect	58	1.08	3	0.0876	1	0.567
Phenylalanine and Tyrosine Metabolism	28	0.52	2	0.0926	1	0.567
Selenoamino Acid Metabolism	28	0.52	2	0.0926	1	0.567
Pterine Biosynthesis	29	0.538	2	0.0984	1	0.567
Citric Acid Cycle	32	0.594	2	0.116	1	0.633
Gluconeogenesis	35	0.649	2	0.135	1	0.697
Phenylacetate Metabolism	9	0.167	1	0.156	1	0.727
Thiamine Metabolism	9	0.167	1	0.156	1	0.727
Pyruvaldehyde Degradation	10	0.186	1	0.171	1	0.764
Histidine Metabolism	43	0.798	2	0.188	1	0.767
Methionine Metabolism	43	0.798	2	0.188	1	0.767
Vitamin K Metabolism	14	0.26	1	0.232	1	0.909
Butyrate Metabolism	19	0.353	1	0.302	1	0.982
Ethanol Degradation	19	0.353	1	0.302	1	0.982
Mitochondrial Electron Transport Chain	19	0.353	1	0.302	1	0.982
Tryptophan Metabolism	60	1.11	2	0.307	1	0.982
Valine, Leucine and Isoleucine Degradation	60	1.11	2	0.307	1	0.982
Riboflavin Metabolism	20	0.371	1	0.315	1	0.982
Pantothenate and CoA Biosynthesis	21	0.39	1	0.328	1	0.982
Carnitine Synthesis	22	0.408	1	0.341	1	0.982

Pathways	Total	Expected	Hits	Raw p	Holm p	FDR
Transfer of Acetyl Groups into Mitochondria	22	0.408	1	0.341	1	0.982
Androstenedione Metabolism	24	0.445	1	0.365	1	0.988
Glycolysis	25	0.464	1	0.377	1	0.988
Tyrosine Metabolism	72	1.34	2	0.391	1	0.988
Mitochondrial Beta-Oxidation of Medium Chain Saturated Fatty Acids	27	0.501	1	0.401	1	0.988
Mitochondrial Beta-Oxidation of Short Chain Saturated Fatty Acids	27	0.501	1	0.401	1	0.988
Mitochondrial Beta-Oxidation of Long Chain Saturated Fatty Acids	28	0.52	1	0.412	1	0.988
Folate Metabolism	29	0.538	1	0.423	1	0.988
Pentose Phosphate Pathway	29	0.538	1	0.423	1	0.988
Amino Sugar Metabolism	33	0.612	1	0.466	1	1
Androgen and Estrogen Metabolism	33	0.612	1	0.466	1	1
Beta-Alanine Metabolism	34	0.631	1	0.477	1	1
Nicotinate and Nicotinamide Metabolism	37	0.687	1	0.506	1	1
Porphyrin Metabolism	40	0.742	1	0.534	1	1
Fatty acid Metabolism	43	0.798	1	0.561	1	1
Steroid Biosynthesis	48	0.891	1	0.602	1	1
Pyrimidine Metabolism	59	1.09	1	0.679	1	1
