# Supplementary material for: APOE genotype influences the gut microbiome structure and function in humans and mice: relevance for Alzheimer’s disease pathophysiology
Source: FASEB J. 2019 Apr 8;33(7):8221–31. doi: 10.1096/fj.201900071R (PMC6593891; doi:10.1096/fj.201900071R)
Supplement: Supplementary file 19 [file fj.201900071R.st7.pdf]

Table S7. Metabolites Set Enrichment associated to age for the faecal metabolites (top 50 pathways).

Pathways	Total	Expected	Hits	Raw p	Holm p	FDR
Ammonia Recycling	32	1.16	7	7.83E-05	0.00767	0.00767
Urea Cycle	29	1.05	6	0.00038	0.0369	0.0186
Glycine and Serine Metabolism	59	2.13	8	0.000777	0.0746	0.0254
Glutamate Metabolism	49	1.77	7	0.00127	0.121	0.0312
Alanine Metabolism	17	0.614	4	0.00246	0.231	0.0481
Aspartate Metabolism	35	1.26	5	0.00687	0.639	0.112
Arginine and Proline Metabolism	53	1.92	6	0.00969	0.892	0.136
Pyruvate Metabolism	48	1.73	5	0.0257	1	0.277
Butyrate Metabolism	19	0.687	3	0.0282	1	0.277
Ethanol Degradation	19	0.687	3	0.0282	1	0.277
Phenylacetate Metabolism	9	0.325	2	0.039	1	0.34
Carnitine Synthesis	22	0.795	3	0.0417	1	0.34
Valine, Leucine and Isoleucine Degradation	60	2.17	5	0.0597	1	0.45
Methionine Metabolism	43	1.55	4	0.0649	1	0.454
Phenylalanine and Tyrosine Metabolism	28	1.01	3	0.0764	1	0.473
Glucose-Alanine Cycle	13	0.47	2	0.0772	1	0.473
Amino Sugar Metabolism	33	1.19	3	0.113	1	0.649
Spermidine and Spermine Biosynthesis	18	0.65	2	0.135	1	0.707
Methylhistidine Metabolism	4	0.145	1	0.137	1	0.707
Warburg Effect	58	2.1	4	0.152	1	0.745
Betaine Metabolism	21	0.759	2	0.174	1	0.774
Glutathione Metabolism	21	0.759	2	0.174	1	0.774
Propanoate Metabolism	42	1.52	3	0.19	1	0.811
Cysteine Metabolism	26	0.939	2	0.241	1	0.922
Biotin Metabolism	8	0.289	1	0.256	1	0.922
Selenoamino Acid Metabolism	28	1.01	2	0.268	1	0.922
Purine Metabolism	74	2.67	4	0.276	1	0.922
Pentose Phosphate Pathway	29	1.05	2	0.282	1	0.922
Thiamine Metabolism	9	0.325	1	0.283	1	0.922
Malate-Aspartate Shuttle	10	0.361	1	0.309	1	0.922
Pyruvaldehyde Degradation	10	0.361	1	0.309	1	0.922

Pathways	Total	Expected	Hits	Raw p	Holm p	FDR
Citric Acid Cycle	32	1.16	2	0.323	1	0.922
Beta-Alanine Metabolism	34	1.23	2	0.35	1	0.922
Phosphatidylethanolamine Biosynthesis	12	0.434	1	0.359	1	0.922
Taurine and Hypotaurine Metabolism	12	0.434	1	0.359	1	0.922
Fatty Acid Biosynthesis	35	1.26	2	0.364	1	0.922
Gluconeogenesis	35	1.26	2	0.364	1	0.922
Tryptophan Metabolism	60	2.17	3	0.371	1	0.922
Ketone Body Metabolism	13	0.47	1	0.382	1	0.922
Thyroid hormone synthesis	13	0.47	1	0.382	1	0.922
Nicotinate and Nicotinamide Metabolism	37	1.34	2	0.39	1	0.922
Phosphatidylcholine Biosynthesis	14	0.506	1	0.405	1	0.922
Vitamin K Metabolism	14	0.506	1	0.405	1	0.922
Histidine Metabolism	43	1.55	2	0.467	1	1
Tyrosine Metabolism	72	2.6	3	0.49	1	1
Mitochondrial Electron Transport Chain	19	0.687	1	0.506	1	1
Catecholamine Biosynthesis	20	0.723	1	0.524	1	1
Riboflavin Metabolism	20	0.723	1	0.524	1	1
Threonine and 2-Oxobutanoate Degradation	20	0.723	1	0.524	1	1
Pantothenate and CoA Biosynthesis	21	0.759	1	0.542	1	1
