# Supplementary material for: APOE genotype influences the gut microbiome structure and function in humans and mice: relevance for Alzheimer’s disease pathophysiology
Source: FASEB J. 2019 Apr 8;33(7):8221–31. doi: 10.1096/fj.201900071R (PMC6593891; doi:10.1096/fj.201900071R)
Supplement: Supplementary file 12 [file fj.201900071R.sf12.pdf]

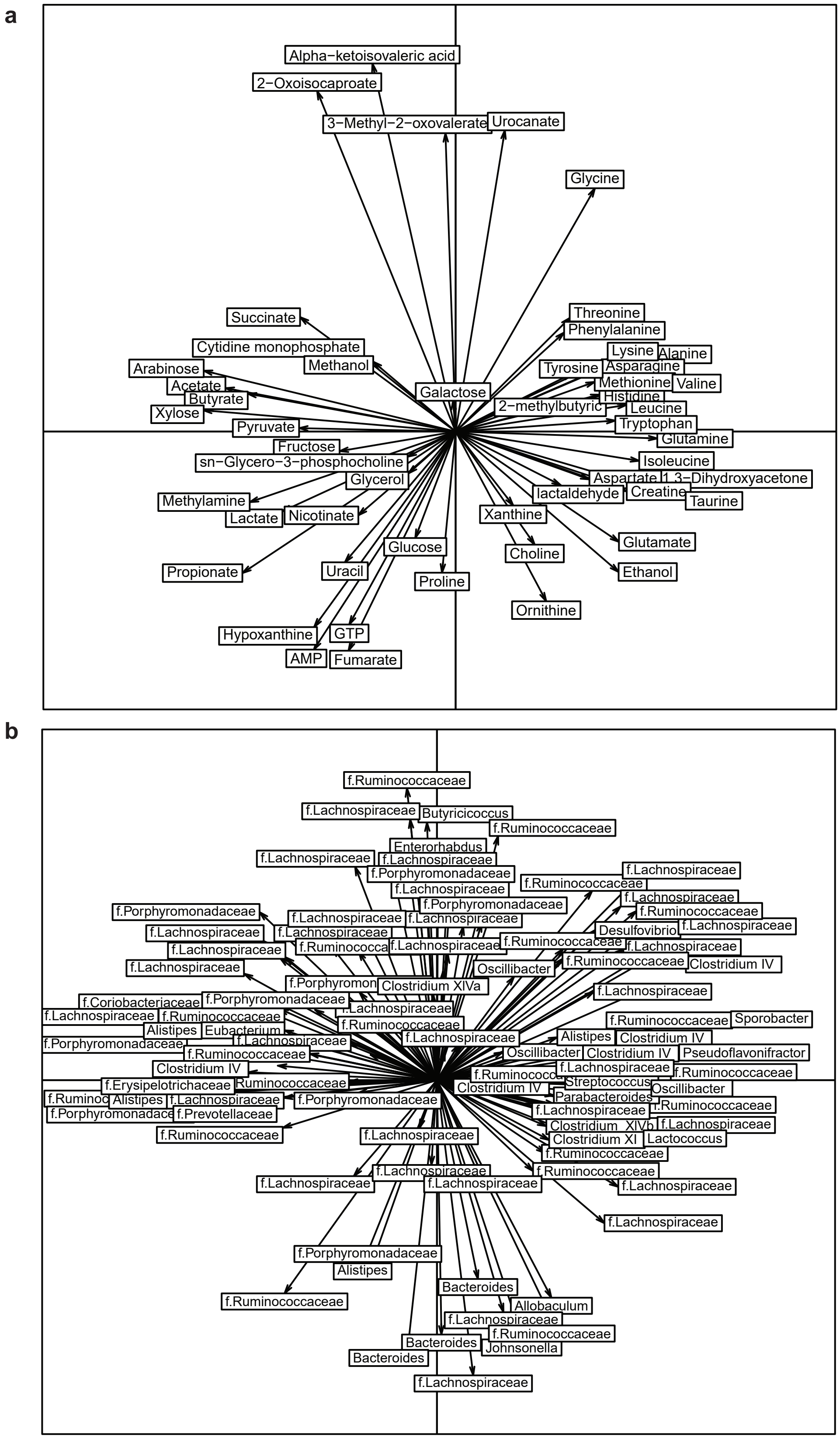

Figure S12. Co-inertia analysis (COIA). Projection of (**a**) metabolites and (**b**) bacterial OTUs labelled with either genus name or family (f.) names for unclassified genera on the first two COIA axes, with the length of arrow indicating the strength of the correlation. There was an agreement between the metabolite abundance and the specific taxon abundance. Only metabolites and OTUs that are highly correlated with corresponding COIA axes are shown.
